# Radiofluorination of a highly potent ATM inhibitor as a potential PET imaging agent

**DOI:** 10.1186/s13550-022-00920-z

**Published:** 2022-08-13

**Authors:** Claudia Rose Fraser, Javier Ajenjo, Mathew Veal, Gemma Marie Dias, Chung Chan, Edward O’Neill, Gianluca Destro, Doreen Lau, Anna Pacelli, Veronique Gouverneur, Rebekka Hueting, Bart Cornelissen

**Affiliations:** 1 Department of Oncology, MRC Oxford Institute for Radiation Oncology, University of Oxford, Old Road Campus Research Building, Roosevelt Drive, Oxford, OX3 7DQ UK; 2 Department of Chemistry, University of Oxford, Oxford, UK; 3 Nuclear Medicine and Molecular Imaging, University Medical Centre Groningen, University of Groningen, Groningen, The Netherlands

**Keywords:** PET, ATM, Cancer, Molecular imaging

## Abstract

**Purpose:**

Ataxia telangiectasia mutated (ATM) is a key mediator of the DNA damage response, and several ATM inhibitors (ATMi) are currently undergoing early phase clinical trials for the treatment of cancer. A radiolabelled ATMi to determine drug pharmacokinetics could assist patient selection in a move towards more personalised medicine. The aim of this study was to synthesise and investigate the first ^18^F-labelled ATM inhibitor [^18^F]**1** for non-invasive imaging of ATM protein and ATMi pharmacokinetics.

**Methods:**

Radiofluorination of a confirmed selective ATM inhibitor (**1**) was achieved through substitution of a nitro-precursor with [^18^F]fluoride. Uptake of [^18^F]**1** was assessed in vitro in H1299 lung cancer cells stably transfected with shRNA to reduce expression of ATM. Blocking studies using several non-radioactive ATM inhibitors assessed binding specificity to ATM. In vivo biodistribution studies were performed in wild-type and ATM-knockout C57BL/6 mice using PET/CT and ex vivo analysis. Uptake of [^18^F]**1** in H1299 tumour xenografts was assessed in BALB/c *nu*/*nu* mice.

**Results:**

Nitro-precursor **2** was synthesised with an overall yield of 12%. Radiofluorination of **2** achieved radiochemically pure [^18^F]**1** in 80 ± 13 min with a radiochemical yield of 20 ± 13% (decay-corrected) and molar activities up to 79.5 GBq/μmol (*n* = 11). In vitro, cell-associated activity of [^18^F]**1** increased over 1 h, and retention of [^18^F]**1** dropped to 50% over 2 h. [^18^F]**1** uptake did not correlate with ATM expression, but could be reduced significantly with an excess of known ATM inhibitors, demonstrating specific binding of [^18^F]**1** to ATM. In vivo, fast hepatobiliary clearance was observed with tumour uptake ranging 0.13–0.90%ID/g after 1 h.

**Conclusion:**

Here, we report the first radiofluorination of an ATM inhibitor and its in vitro and in vivo biological evaluations, revealing the benefits but also some limitations of ^18^F-labelled ATM inhibitors.

**Supplementary Information:**

The online version contains supplementary material available at 10.1186/s13550-022-00920-z.

## Introduction

Ataxia telangiectasia mutated (ATM) is a key mediator of the DNA damage response with a crucial role in maintaining genomic integrity, making it significant in the study and therapy of cancer. When ATM is activated in the presence of double-stranded DNA breaks (DSBs) resulting from genomic instability, replication stress, or external stimuli such as radio- or chemotherapy, it undergoes monomerisation and autophosphorylation at S1981. Its central role in DNA damage repair signalling has led to a variety of ATM inhibitors (ATMi) being developed to reduce ATM enzymatic activity, restricting repair of harmful lesions and cell cycle arrest, resulting in mitotic catastrophe and cell death (Table 1) [1, 2]. Although there are a handful of ATM inhibitors currently undergoing clinical trials, none have been approved for use in patients yet.

**Table 1:** ATM inhibitors under preclinical and clinical investigation

Name	Structure	ATM IC_50_ (nM)	Status/Key findings
AZD1390		0.78	Phase I clinical trial for brain cancer in combination with radiotherapy [16] Phase I clinical trial for NSCLC in combination with radiotherapy [17]. Phase I clinical trial completed to assess brain PET imaging with ^11^C-labelled AZD1390 (labelled in the starred position) [5, 6]
AZD0156		0.58	Phase I clinical trial for advanced solid tumours, alone and in combination with other agents [18, 19]
M4076		0.2	Phase I clinical trial for advanced solid tumours [20, 21]
M3541		0.5	Completed phase I clinical trials for solid tumours in combination with radiotherapy [22]
KU60019		6.3	Preclinical *in vitro* evaluation of KU60019 in combination with CHK2 inhibitor, CX4945 [23, 24]
KU55933		13	First specific ATM inhibitor, demonstrated sensitisation of cells to ionising radiation and topoisomerase inhibitors [25]
KU59403		3	Preclinical investigation in combination with topoisomerase inhibitors and improved pharmacologic properties over previous KuDOS inhibitors [11]
CP466722		410	Demonstrated rapid and reversible inhibition of ATM *in vitro*, revealing that only transient ATM inhibition is require for cell sensitisation to ionising radiation [26, 27]

The study of ATM both mechanistically and as a therapeutic target has led to the development of a range of techniques to visualise ATM, recently reviewed [3]. Investigations into the activation mechanisms of ATM have historically used classical techniques such as immunoblotting to identify ATM activation and the effects on downstream targets of ATM, aided by the use of increasingly selective ATM inhibitors. Due to the complexity of ATM mechanisms, the study of proteins associated with ATM has also been used to assess its activity indirectly. One elegant example is the use of PET with ^18^F-labelled 1-(2′-deoxy-2′-^18^F-fluoroarabinofuranosyl) cytosine ([^18^F]FAC) to image the expression of deoxycytidine kinase (dCK), an enzyme that is phosphorylated by ATM, activating its role in nucleotide salvage for DNA damage repair [4]. More recently, direct imaging of the biodistribution of an ATM inhibitor using a ^11^C-labelled version, [^11^C]AZD1390, has demonstrated that particular inhibitor’s ability to cross the blood–brain barrier in a phase I clinical trial [5, 6].

Radiolabelled analogues of investigational drugs add enormous value for the study of pharmacokinetics, can aid preclinical development, and act as companion biomarkers assisting patient stratification [7]. PET/SPECT imaging with these radiopharmaceuticals can be used to non-invasively assess tumour/tissue uptake and aid dose calculations and scheduling for each patient prior to treatment with the therapeutic, assisting the move to more personalised medicine. [^11^C]AZD1390 demonstrated some of these benefits; however, the relatively short half-life of ^11^C (20 min) does limit the clinical applicability of ^11^C-labelled small molecules.

**Fig. 1:**
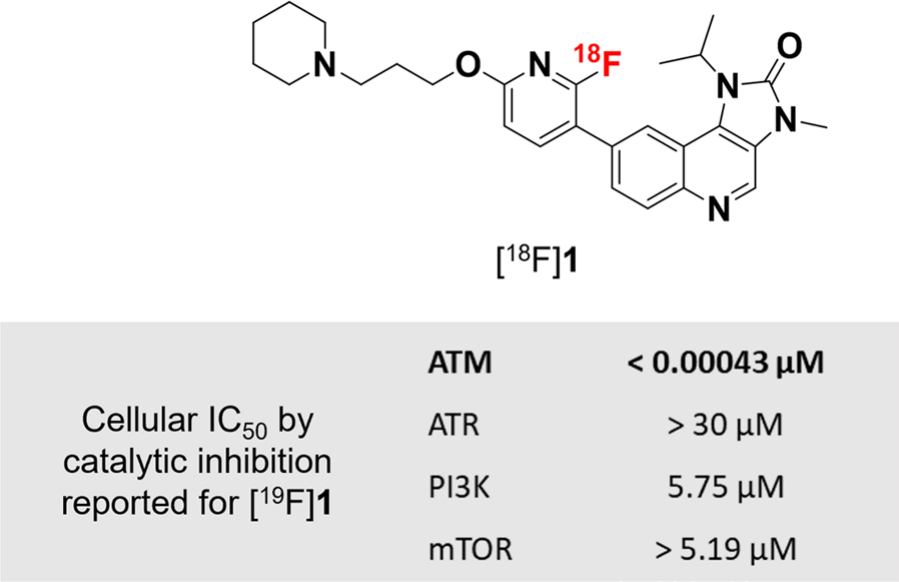
Target compound [^18^F]**1** and reported inhibitory data [8]

The need for an ATM inhibitor labelled with a radionuclide that correlates with the pharmacokinetics of the inhibitor itself led to this work to create the first ^18^F-labelled ATM inhibitor, utilising fluorine-18 as a radioisotope with a longer decay half-life of 110 min. The compound was chosen from a series of ATM inhibitors reported by Baalam et al*.* that includes a number of highly potent and selective molecules containing fluoride moieties in their structures [8]. Based on the structures and ATM inhibition data reported in this series (with regard to ease of synthesis, likelihood of ^18^F-labelling, and ATM selectivity), we developed a synthetic route to access an ^18^F-labelled ATM inhibitor, [^18^F]**1** (Fig. 1). Herein, we report the synthesis of a nitro-precursor molecule **2** that is suitable for nucleophilic radiofluorination to afford [^18^F]**1**, and biological assessment of [^18^F]**1** in vitro and in vivo*,* investigating its potential as an ATM PET imaging agent.

## Methods

### Chemical synthesis

**Fig. 2:**
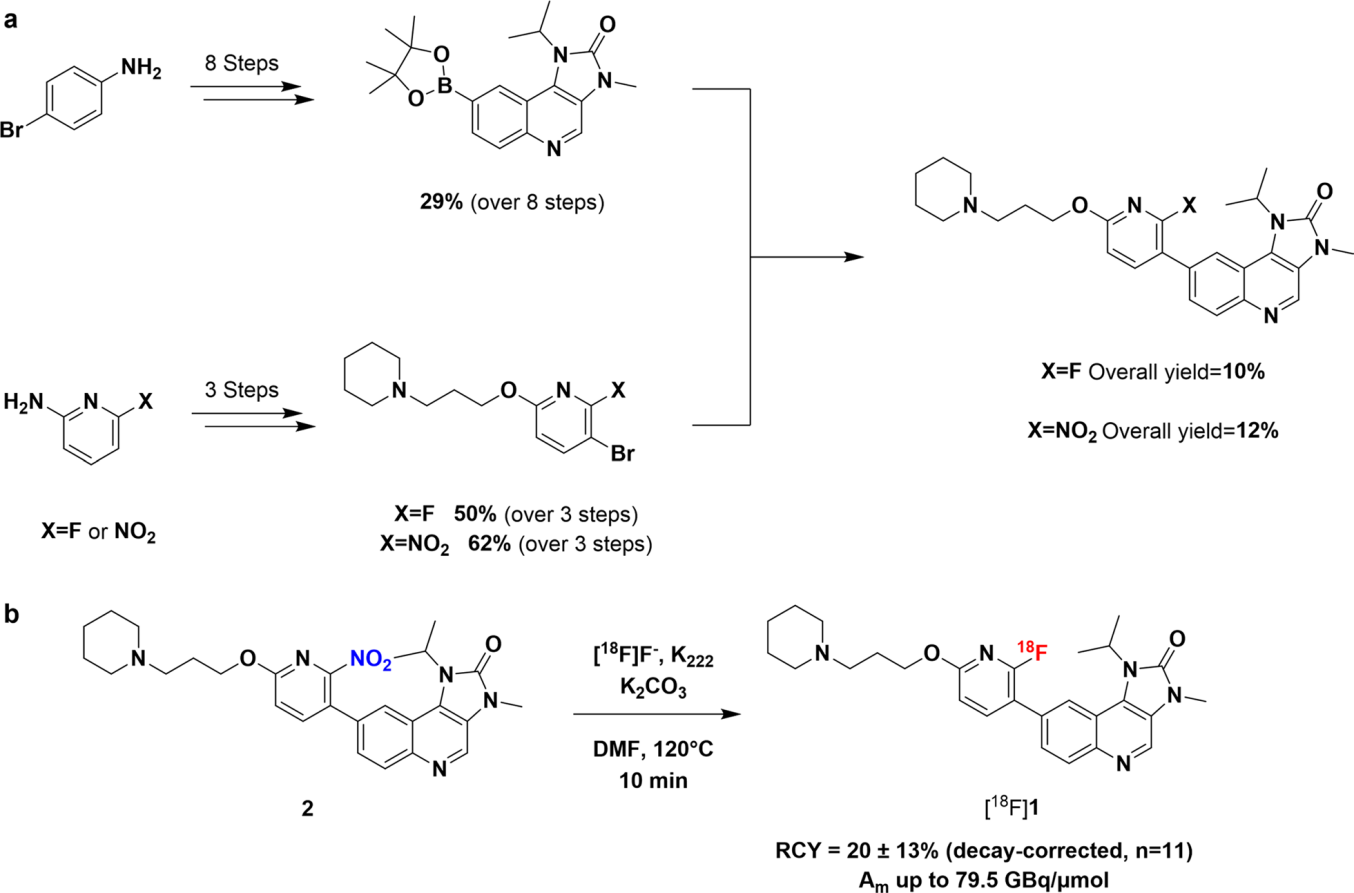
**a** Synthetic pathway to reference [^19^F]**1** and nitro-precursor **2**. **b** Radiosynthesis of [^18^F]**1** from **2**

The synthesis of the non-radioactive ^19^F version of compound **1** followed methods reported by patent [8], with minor modifications as detailed in the supporting information (Fig. 2; Additional file 1: Fig. S1). The synthesis of nitro-precursor **2** followed the same route as for [^19^F]**1**. Full synthetic details are available in the supporting information.

### Radiosynthesis

Radiosynthesis of [^18^F]**1** was achieved through nucleophilic aromatic substitution (S_N_Ar) of **2** (1 mg) with [^18^F]fluoride (5–12 GBq) dried azeotropically with acetonitrile in the presence of K_2_CO_3_ (6 mg) and K_2,2,2_ (30 mg) reacting for 10 min at 120 °C in DMF (Fig. 2). HPLC purification of [^18^F]**1** was performed using a Luna® 10 µm C18(2) 100 Å 250 × 10 mm semi-preparative column (Phenomenex) in 25:75 acetonitrile/0.1% formic acid aqueous buffer, followed by reformulation in 10% DMSO in 0.9% saline solution for in vitro and in vivo studies. Manual radiolabelling reactions were performed using aliquots of [^18^F]fluoride (approx. 200 MBq) azeotropically dried with acetonitrile with addition of K_2_CO_3_ (1 mg) and K_2,2,2_ (5 mg) before the subsequent labelling reactions. The radiosynthesis was fully automated on an Eckert & Ziegler Modular-Lab system (full description of setup available in the supporting information, Additional file 1: Figs. S2–S5).

### In vitro evaluation

H1299 non-small cell lung cancer cells, either wild type or stably transfected with short hairpin RNA for ATM (shATM) or GFP (shGFP, as a control) expression knockdown, were kindly gifted by Dr Anderson Ryan at the University of Oxford [9]. All cells were cultured at 37 °C and 5% CO_2_ in Dulbecco’s modified Eagle medium (DMEM) supplemented with 10% foetal bovine serum (FBS) (Gibco, USA), 2 mM L-glutamine, 100 units/mL penicillin, and 100 µg/mL streptomycin (PSG) (Sigma-Aldrich); knockdown cell lines were additionally supplemented with 0.5 μg/mL puromycin dihydrochloride (Merck Life Science, UK). Cells were originally purchased from ATCC and were authenticated by the provider and by STR profiling. Cells were tested regularly for the absence of mycoplasma contamination and were cultured for a maximum of 20 passages after resuscitation from liquid nitrogen storage.

Western blot analysis was performed to compare the total ATM expression between cell models, in addition to expression of phospho-ATM^S1981^ (pATM), phospho-KAP1^S824^ (pKAP1), and phospho-CHK2^T68^ (pCHK2) expression to assess the effect of irradiation and ATM inhibition on ATM catalytic activity. Cells were irradiated (4 Gy; 0.75 Gy/min) or sham-irradiated and incubated at 37 °C for 1 h before harvesting cells for lysis and blotting (full details in supporting information). Where required, inhibitors were added to cells at the stated concentration from DMSO stock solutions (reaching no greater than 10% DMSO final concentration) with incubation for 1 h prior to irradiation/sham-irradiation. The following antibodies and dilutions were used in this work: phospho-ATM S1981 (ab81292, 1:5000, Abcam); phospho-KAP1 S824 (ab133440, 1:10,000, Abcam); phospho-CHK2 T68 (#2661, 1:1000, Cell Signalling Technologies); Total ATM (ab32420, clone [Y170], 1:500, Abcam); Total KAP1 (ab10483, 1:1000, Abcam); Total CHK2 (05-649, 1:1000, Merck Millipore); HRP-conjugated Tubulin (ab197740, clone [YL1/2], 1:4000, Abcam); HRP-conjugated β-Actin (clone 13E5, 1:1000, Cell Signalling Technologies), HRP-conjugated anti-rabbit secondary (HAF008, 1:1000, R&D Systems); and HRP-conjugated anti-mouse secondary (HAF007, 1:1000, R&D Systems). All blots were repeated on at least two separate occasions with new cell lysates for validation.

To assess cellular uptake of [^18^F]**1**, aliquots of H1299 shATM or shGFP cells were plated with 1 × 10^5^ cells per well in 24-well plates in 500 μL complete growth medium and incubated overnight to allow cells to adhere. Cell culture medium was removed, cells washed with PBS and media replaced with DMEM (no additives). Cells were irradiated (4 Gy; 0.75 Gy/min) or sham-irradiated and incubated for 1 h before addition of [^18^F]**1**, followed by incubation at 37 °C and 5% CO_2_ for the indicated time. Supernatant cell culture medium was collected, and cells were washed twice with PBS, after which they were lysed with 0.1 M sodium hydroxide (200 μL) for 30 min. The amount of ^18^F-activity associated with each fraction was determined using a Wizard2 Automatic Gamma Counter (PerkinElmer).

Retention assays followed the same procedure with a 1-h incubation of cell with [^18^F]**1**, but after culture medium was aspirated and cells washed with PBS (2 × 500 μL), it was replaced (500 μL DMEM) and cells incubated at 37 °C and 5% CO_2_ for the desired time. The amount of ^18^F-activity retained in cells was then determined as above.

Blocking studies followed the same procedure as uptake with 1 h tracer incubation; however, the replacement media contained the blocking agent at the indicated concentrations in no greater than 10% DMSO, and a DMSO control was also run in parallel at the highest DMSO concentration used, followed by incubation at 37 °C and 5% CO_2_ for 1 h before irradiation.

### In vivo evaluation

All animal procedures were performed in accordance with the UK Animals (Scientific Procedures) Act 1986 and with local ethical committee approval. Mice were bred from parent C57BL/6 J mice heterozygous for a targeted mutation in the ATM^tm1Awbl^ allele to produce ATM-null (ATM^−/−^) mice (Jackson laboratory). Wild-type (ATM^+/+^) mice from this breeding program were used as controls. In a separate study, tumour xenografts were created in BALB/c *nu*/*nu* mice (8 weeks old) via subcutaneous injection of H1299 wild type, shATM knockdown, or shGFP knockdown cells in a 1:1 PBS/Matrigel® formulation on both sides of the hind flank. Total tumour volumes were allowed to reach an average of 600 mm^3^ (range 330–930 mm^3^) before imaging and/or biodistribution studies (approx. 6–10 weeks after inoculation).

Animals were anaesthetised by 4% isoflurane gas (0.5 L/min O_2_) and maintained at approx. 2% and 37 °C throughout the imaging session. C57BL/6 ATM^+/+^ and ATM^−/−^ mice were administered [^18^F]**1** (83 ng; 0.5–4.6 MBq, molar activity range of 3.2–24.2 GBq/μmol with a standard deviation of 6.5 GBq/μmol) via intravenous bolus injection in the lateral tail vein. Xenograft-bearing BALB/c *nu*/*nu* mice were administered [^18^F]**1** (0.5 μg; 0.7–9.6 MBq, molar activity range of 0.62–9.8 GBq/μmol with a standard deviation of 3.0 GBq/μmol) via intravenous bolus injection to the lateral tail vein. PET/CT scanning was completed using a VECTor4CT scanner (MILabs, Utrecht, the Netherlands) for 1 h and was immediately followed by culling, dissection, and gamma counting of selected organs. Tumour tissue was immediately flash frozen in liquid nitrogen and mounted in OCT before producing 10 μm thick sections for autoradiography and H&E staining. Alternatively, frozen tumour tissue was macerated and lysed in RIPA buffer for western blot analysis (full details available in the supporting information). Reconstruction of both CT and PET images was performed with the MILabs reconstruction analysis using a γ-ray energy window of 467–571 keV (background weight 2.5%), 0.8 mm^3^ voxel size, 8 subsets, and 5 iterations using the manufacturer’s SROSEM reconstruction type. PET images were each registered to CT, attenuation corrected, calibrated by imaging a phantom containing a fluorine-18 standard solution, and analysed using PMod software package (version 3.807, PMOD Technologies).

### Statistical analysis

Statistical analysis was performed using GraphPad Prism v9 (GraphPad Software, San Diego, CA, USA). In each case, a 1-way or 2-way ANOVA was performed with multiple comparisons using Šidák’s or Tukey’s test, with significance denoted as *p < 0.05, **p < 0.01, ***p < 0.001, ****p < 0.0001, or ns *p* > 0.05.

## Results

The synthesis of reference compound [^19^F]**1** was completed with an overall yield of 10% over 12 steps. Nitro-precursor **2** was prepared with a yield of 12% over 12 steps (Fig. 2; Additional file 1: Fig. S1).

Reaction condition screening during manual radiofluorination of precursor **2** revealed the best radiochemical conversion was obtained in DMF at 120 °C for 10 min. The automated radiosynthesis of [^18^F]**1**, performed on an Eckert & Ziegler Modular-Lab, was completed in 80 ± 13 min with a radiochemical yield of 20 ± 13% (decay-corrected), radiochemical purity > 99%, and molar activity up to 79.5 GBq/μmol (*n* = 11) (Fig. 2; Additional file 1: Figs. S2–S5).

**Fig. 3:**
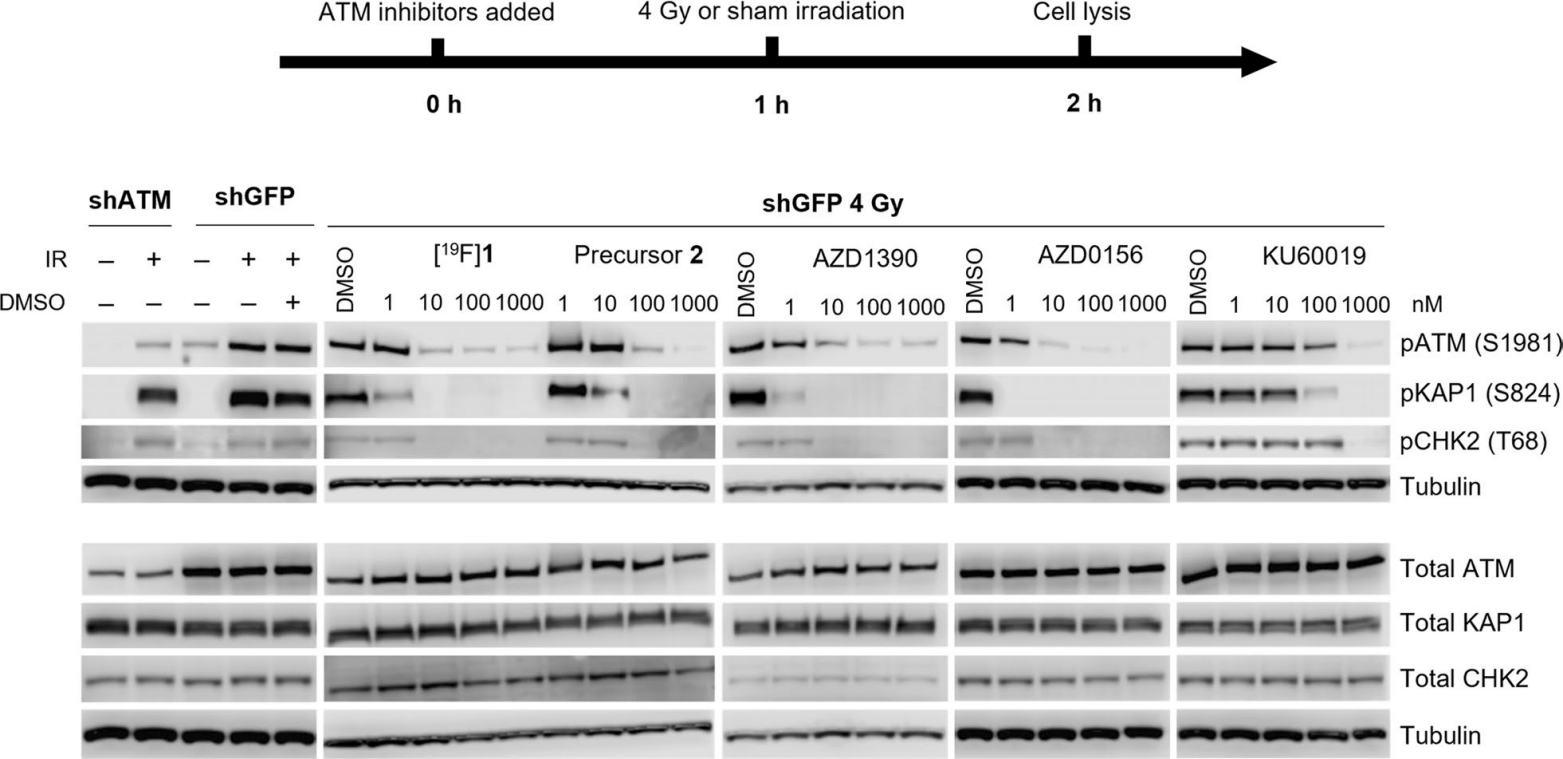
Higher ATM expression is observed in H1299 shGFP knockdown cells compared to H1299 shATM knockdowns. Irradiation of cells (4 Gy) causes an increased expression of pATM, pKAP1, and pCHK2. A decrease in expression of pATM and downstream targets pKAP1 and pCHK2 is observed following treatment of shGFP (4 Gy) cells with different ATM inhibitors

Western blot analysis confirmed a reduced total ATM expression in the shATM knockdown H1299 cells compared to the shGFP knockdown H1299 cells, and reduced phospho-ATM^S1981^ (pATM) expression in irradiated shATM cells compared to irradiated shGFP cells. In irradiated shGFP knockdown cells, ATM inhibition with known ATM inhibitors AZD1390, [^19^F]**1**, AZD0156, KU60019, as well as precursor **2**, were confirmed by a decrease in pATM expression, as well as expression of downstream targets of pATM, pKAP1, and pCHK2, relative to a DMSO control (Fig. 3).

**Fig. 4:**
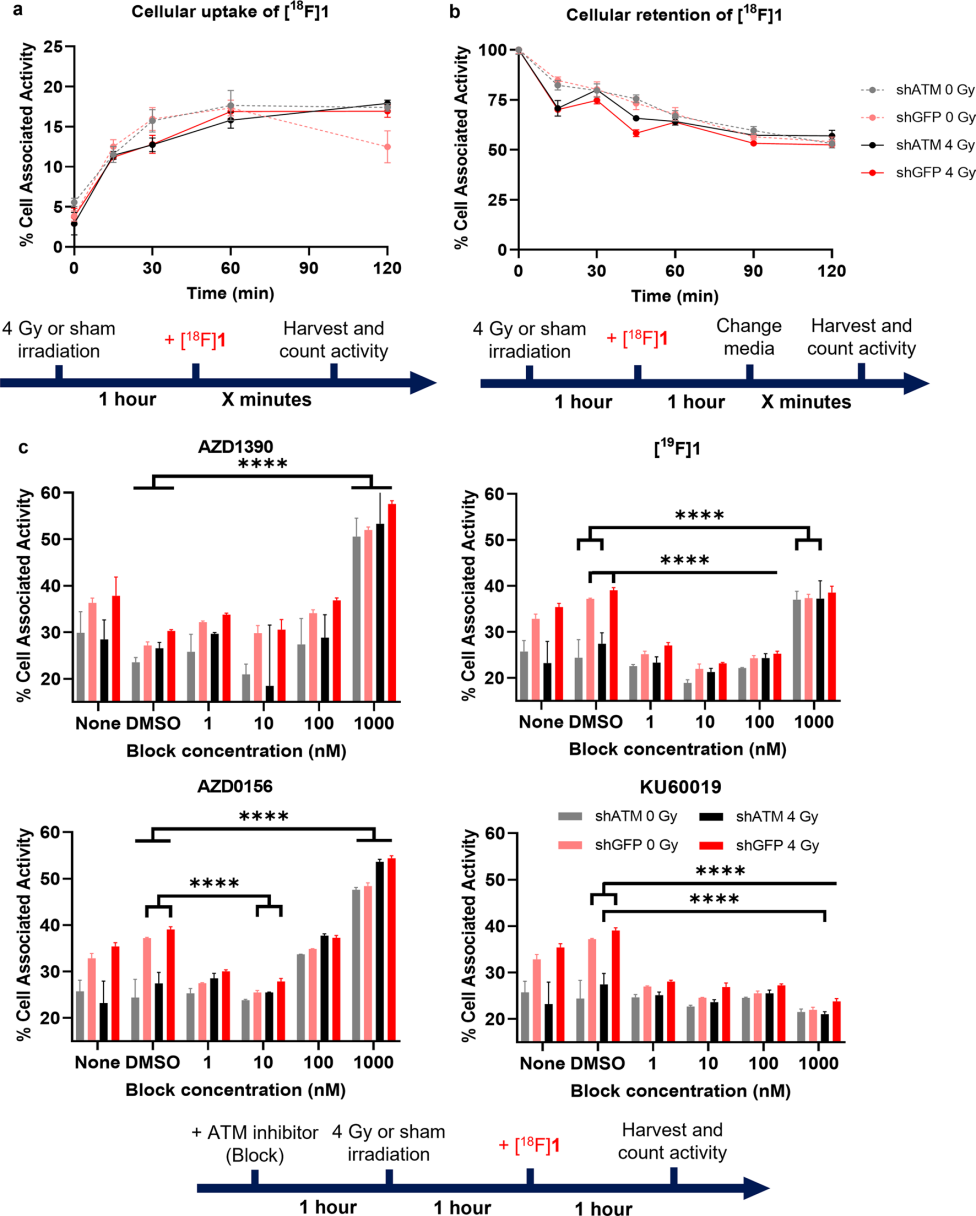
**a** Cell uptake and **b** retention of [^18^F]**1** in H1299 shATM and shGFP cells. **c** Cell-associated activity in H1299 shATM and shGFP cells after pre-treatment with a range of ATM inhibitors (block)

Uptake of [^18^F]**1** in cells showed an increase in cell-associated activity over 1 h followed by a plateau. Retention of [^18^F]**1** in the same cells revealed a gradual decrease in cell-associated activity of approximately 50% over 2 h. No significant differences between cell-associated activity were observed between the different cell lines/irradiation conditions. Nonetheless, significant blocking was observed with increasing concentrations of the ATM inhibitor KU60019, demonstrating some specific binding of [^18^F]**1** to ATM and revealing high levels of non-specific uptake. Surprisingly, addition of some known ATM inhibitors AZD1390, AZD0156, or [^19^F]**1** resulted in an initial blocking effect at lower concentrations of the inhibitor (0.1–10 nM), but an increase in cell-associated activity at higher concentrations (Fig. 4).

**Fig. 5:**
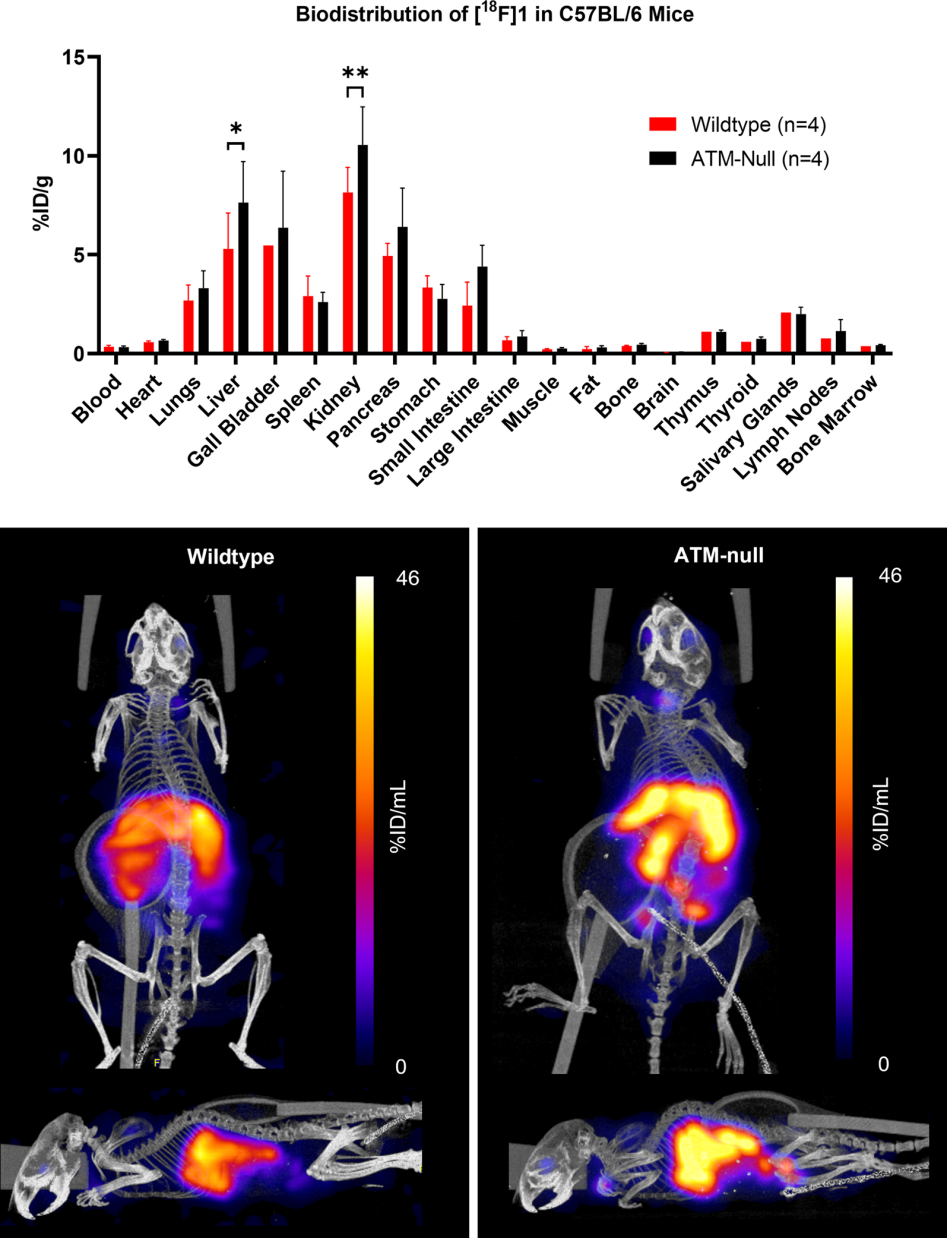
Biodistribution of [^18^F]**1** in C57BL/6 ATM wild type and ATM-knockout mice, 1 h after intravenous administration. Below are shown representative PET/CT MIP images demonstrating the hepatobiliary uptake of tracer in both wild type and ATM-null mice

PET/CT imaging and ex vivo biodistribution of [^18^F]**1** in wild type and ATM-null C57BL/6 mice 1 h after intravenous administration revealed hepatobiliary clearance (Fig. 5). Significant differences in uptake between the two mice were only observed in the kidneys and liver. Challenges due to [^18^F]**1** adhering to the walls of long cannula lines during intravenous injection meant that short cannula lines were necessary to administer the dose, resulting in a delayed start of dynamic PET imaging (starting approx. 10 min after injection of the tracer) by which point tissue activity concentrations remained near constant, preventing pharmacokinetic analysis.

**Fig. 6:**
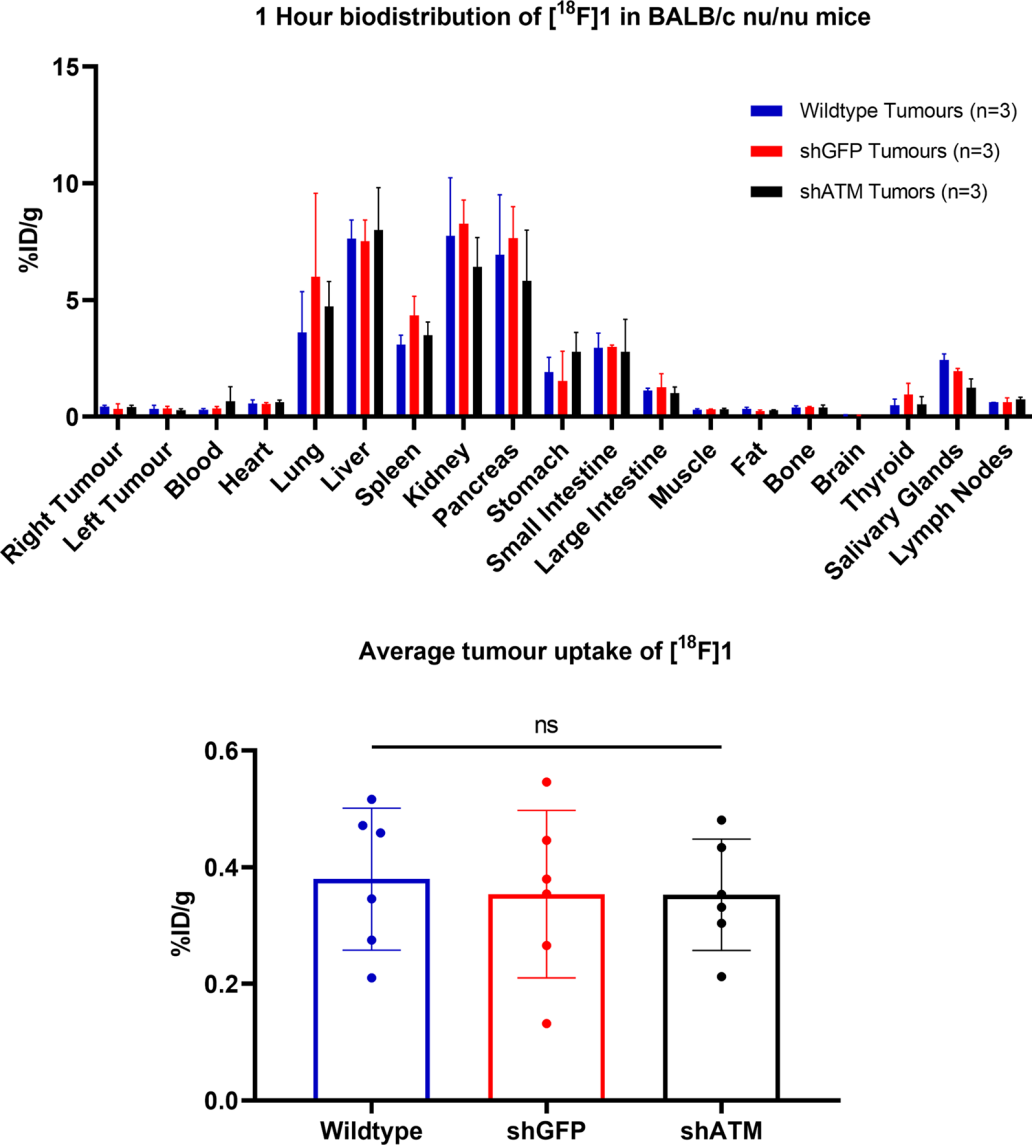
In vivo biodistribution of [^18^F]**1** in BALB/c nu/nu mice bearing subcutaneous H1299 wild type, shATM, or shGFP xenografts

Biodistribution of [^18^F]**1** in BALB/c *nu*/*nu* mice was comparable to C57BL/6 mice. Uptake in H1299 tumour xenografts was measured as 0.38 ± 0.12%ID/g (wild type), 0.35 ± 0.14%ID/g (shGFP), and 0.35 ± 0.10%ID/g (shATM) (Fig. 6). No significant differences were observed in tumour uptake between the different tumour types, reflecting our in vitro results, despite confirmation of xenograft ATM knockdown status by ex vivo western blot (Additional file 1: Fig. S6). Autoradiography of tumour sections revealed heterogeneous uptake across the xenografts that was not specific to tumour type. H&E staining of the same tumour sections revealed regions of tissue lacking nuclei (possibly suggesting necrosis) that matched autoradiography low-signal regions of the tumour, indicating uptake of the tracer only in viable cells (Additional file 1: Fig. S7). The few tumours revealed to have necrotic centres did not have significantly different uptake (%ID/g) compared to the more viable tumours. Autoradiography analysis of viable cell regions (i.e. excluding areas of necrosis) revealed no significance in %ID/cm^2^ between xenograft type (Additional file 1: Fig. S8).

## Discussion

ATM inhibitors are an up-and-coming treatment for cancer. While there are four inhibitors currently undergoing human trials (Table 1), none are currently approved for clinical use. A host of preclinical studies have demonstrated the potential of these inhibitors for cancer treatment, most in combination with other DNA damaging therapies [10, 11]. However, many drugs fail to reach or progress through this vital stage of drug development due to lack of understanding of the drug efficacy. Studying the pharmacokinetics of experimental drugs provides vital information that can aid the transition into clinical evaluation. Drug pharmacokinetics are generally measured through the sampling of blood, urine, and tissue biopsies over time, which can be invasive and cannot reveal drug biodistribution across the whole body or in heterogeneous tissues, or indeed the tumour target distribution.

The use of nuclear imaging can provide a clearer picture of drug pharmacokinetics across the whole body non-invasively with the use of radiolabelled drug analogues [7]. This technique can assist in designing treatment plans for patients (e.g. calculating drug dosage), but can also assist the clinical development of novel drugs. A radiolabelled ATM inhibitor promises to visualise and quantify drug distribution, while also having the potential to give insight into ATM tissue distribution. The previously reported [^11^C]AZD1390 has shown in clinical studies how radiopharmaceuticals can aid these measurements; however, the short half-life of ^11^C reduces the scope of using ^11^C-labelled drug variants due to the requirement of on-site cyclotrons in hospitals. Where the radiochemistry is available and able to retain target binding properties, the use of ^18^F-radiotracers can overcome this issue. Hence, we radiofluorinated potent ATM inhibitor **1** as a tool to investigate ATM PET imaging.

Our in vitro model system demonstrated the ability of **1** to inhibit ATM activity through inhibition of ATM Serine-1981 phosphorylation and the phosphorylation of its downstream targets, KAP1 and CHK2. This showed **1** to have ATM inhibitory properties comparable to other ATM inhibitors such as AZD1390, AZD0156, and KU60019, confirming its reported sub-nM IC_50_ value of ATM inhibition [8]. This data indicates that **1** binds to and inhibits ATM in live cells and reinforces the proposal that [^18^F]**1** could be used to image ATM.

While some specific binding to ATM of [^18^F]**1** was observed, as evident from statistically significant reductions of cell-associated activity after exposure to an excess of the ATM inhibitor KU60019 (*p* < 0.0001), high levels of non-ATM specific cell association were also revealed. This relatively low level of ATM specific uptake relative to the non-specific uptake is likely due to the innately low cellular expression of ATM [12–14] which may lead to only slight observable changes in [^18^F]**1** uptake, in addition to the highly lipophilic nature of the inhibitor which aids passive transport of the compound into the cell. This low ratio of specific to non-specific binding may be masking any differences in uptake that are expected between cell lines with highly varying ATM expression levels, as well as the effects of irradiation (and therefore increased expression of active, phosphorylated ATM) on the uptake of [^18^F]**1**. This result demonstrates the limits of [^18^F]**1** for mechanistic studies of ATM.

Surprisingly, blocking [^18^F]**1** uptake with high molar excess (1000 nM) of the ATM inhibitors AZD1390, AZD0156, or [^19^F]**1** resulted in an increase in cell-associated activity of [^18^F]**1**, compared to a DMSO control, despite demonstration by western blot that total ATM protein levels were not affected by the same doses of the blocking agents. Since ATM expression levels remain constant in these blocking conditions, it is not yet clear what caused this effect. Quinoline-based ATM inhibitors are highly lipophilic compounds, so it may be hypothesised that at high concentrations the blocking compounds could be altering cell membrane permeability, increasing cellular infiltration of [^18^F]**1**. An alternative hypothesis could be that the high blocking concentrations are binding to and blocking drug efflux transporter proteins that expel compound **1**, meaning that [^18^F]**1** becomes trapped in the cell non-specifically. Similarly, the excess blocking agents may be impacting the expression of other kinases unrelated to ATM expression, resulting in increased off-target specific binding of [^18^F]**1.** Further work is needed to determine the binding affinity of [^19^F]**1** to known drug efflux proteins and/or kinases to investigate this theory, and the potential for [^18^F]**1** to image proteins other than ATM.

Although murine ATM is different to human ATM [15], it retains much of the same structure and many of the same phosphorylation sites. We were able to demonstrate murine ATM inhibition by **1** in CT26 murine colorectal cancer cells (and reduction of downstream target pKAP1) using western blot, indicating binding of **1** to murine ATM (Additional file 1: Fig. S9). In vivo, the comparison in biodistribution of [^18^F]**1** in wild type versus ATM-null mice showed significant differences in the liver and kidney uptake (Additional file 1: Fig. 5). Given the observed hepatobiliary and renal clearance (demonstrated by high uptake in the liver, small intestine, and kidneys), it may be hypothesised that the lower uptake in these organs of the wild-type mice could be due to an ‘ATM sink’ effect, where there may be increased specific binding of [^18^F]**1** to ATM across the rest of the body, slowing the hepatobiliary clearance of the tracer (therefore reducing hepatobiliary organ uptake of [^18^F]**1**) relative to the ATM-null mice. Alternatively, this difference could be due to fundamental differences in metabolism between the two mouse strains, i.e. ATM wild type vs. ATM-null. It should be noted that the ATM-null mice were of smaller size and weight than wild-type mice, although ex vivo organ weight as a percentage of body weight was comparable.

Similar hepatobiliary clearance of [^18^F]**1** was observed in xenograft-bearing BALB/c *nu*/*nu* mice, but there was no significant difference between uptake in any of the organs or tumours across the different tumour models (Additional file 1: Fig. 6). This lack of differential uptake in the H1299 tumour xenografts mimics our in vitro uptake results in the same cells, indicating that the use of a radiolabelled ATMi for imaging ATM status may be challenging in a clinical setting, but still demonstrates the usefulness of [^18^F]**1** to non-invasively measure whole body drug biodistribution and quantify tumour-drug uptake.

## Conclusions

To the best of our knowledge, radiolabelled compound [^18^F]**1** is the first reported radiofluorinated ATM inhibitor. We were able to demonstrate the cellular uptake of [^18^F]**1** in vitro and revealed the challenges in determining specific binding to ATM, likely due to low ATM expression in cells in comparison with a high non-specific uptake. In vivo, no significant difference in uptake between tumour xenografts expressing different levels of ATM was observed, but we were able to demonstrate whole body drug biodistribution and the hepatobiliary clearance of [^18^F]**1**. Taken together, these results show the potential challenges in using radiotracers to assess ATM status but highlight the potential benefit of developing similar radiofluorinated ATM inhibitors for the non-invasive assessment of drug pharmacokinetics and tumour distribution.

## Supplementary Information

**Additional file 1:** Supplementary information.

## Data Availability

The datasets used and/or analysed during the current study are available from the corresponding author on reasonable request.
