# Supplementary material for: Radiofluorination of a highly potent ATM inhibitor as a potential PET imaging agent
Source: EJNMMI Res. 2022 Aug 13;12:50. doi: 10.1186/s13550-022-00920-z (PMC9375819; doi:10.1186/s13550-022-00920-z)
Supplement: Supplementary file 1 — Additional file 1: Supplementary information. [file 13550_2022_920_MOESM1_ESM.pdf]

Radiofluorination of a highly potent ATM inhibitor as a potential PET imaging agent

Claudia Fraser[1†], Javier Ajenjo[1†], Mathew Veal[1], Gemma Dias[1], Chung Chan[1], Edward O'Neill[1], Gianluca Destro[1,2], Doreen Lau[1], Anna Pacelli[1], Veronique Gouverneur[2], Rebekka Hueting[1], Bart Cornelissen[1,3]*

[1] MRC Oxford Institute for Radiation Oncology, Department of Oncology, University of Oxford, Oxford, UK

[2] Department of Chemistry, University of Oxford, Oxford, UK

[3] Nuclear Medicine and Molecular Imaging, University Medical Centre Groningen, University of Groningen, Groningen, The Netherlands

[†] These authors contributed equally to this study

Corresponding author:

Professor Bart Cornelissen, Associate Professor
University of Oxford
Oxford Institute for Radiation Oncology
Department of Oncology
Old Road Campus Research Building
Roosevelt Drive Oxford
OX3 7DQ
UK
bart.cornelissen@oncology.ox.ac.uk

Contents

1. Chemical synthesis
2. Radiosynthesis automation
3. Supplementary figures
4. Western blot procedure
5. H&E procedure
6. NMR Spectra of Novel Compounds
7. Supporting information references

1. Chemical synthesis

All reagents used in the synthesis of compound ^{19}F-**1** and **2** were purchased from commercial sources: Sigma Aldrich, Merck, Alfa Aesar, Fluorochem, Across Organics, Manchester Organics, Fisher Scientific. Reactions were monitored characterised by silica thin layer chromatography (TLC), liquid chromatography mass spectrometry (LCMS, Waters HPLC and Acquity QDa Mass detection system), and NMR (400 MHz Bruker Ascend™). Novel compounds were characterised by ^{1}H, ^{13}C, ^{19}F NMR and high-resolution mass spectrometry (HRMS) on a Thermo Exactive High-Resolution Orbitrap FTMS using the HESI-II probe for positive electrospray ionization (ESI^{+}).

Full synthesis schematic

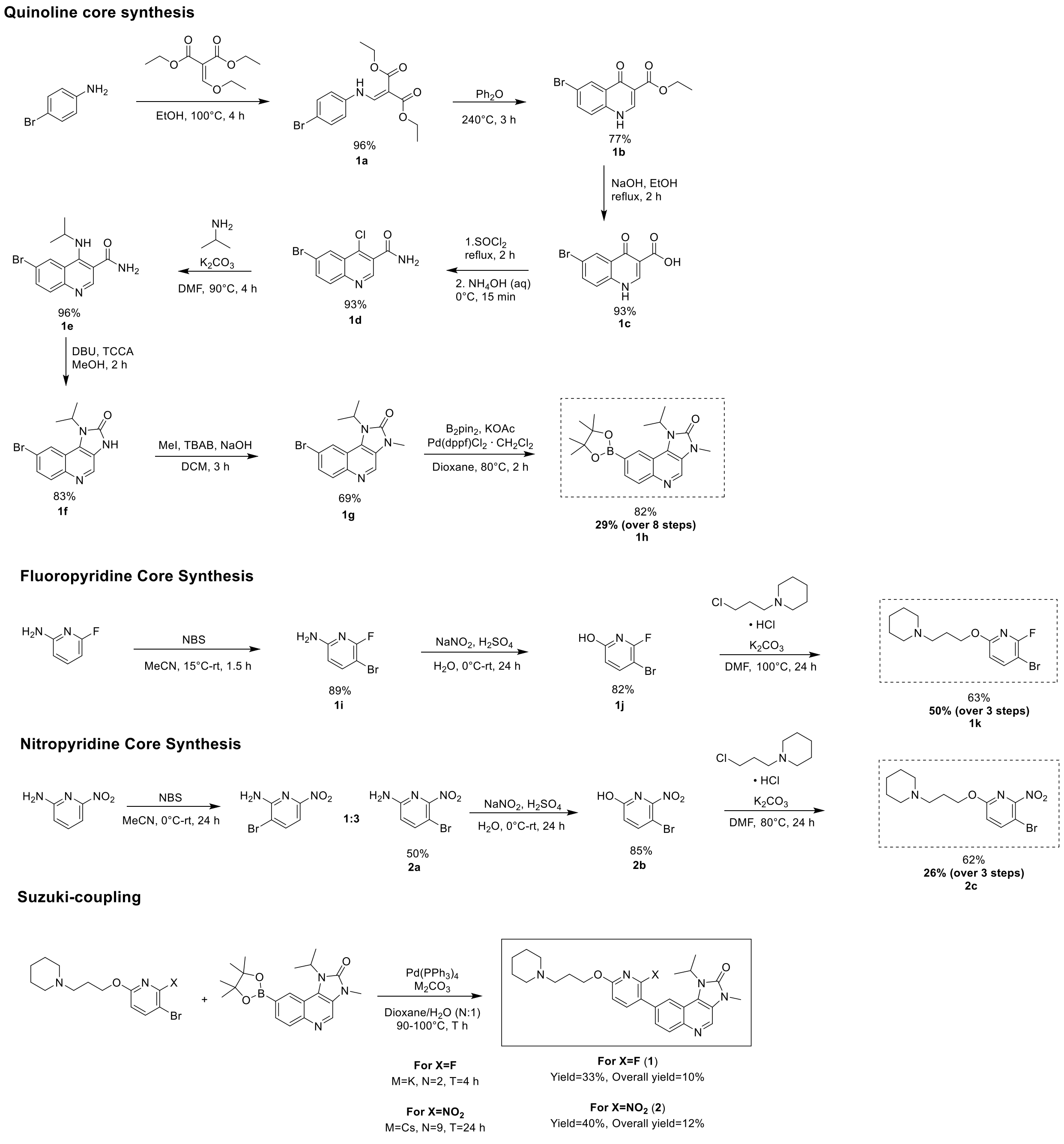

Supplementary Fig. S1 Full scheme for synthesis of reference compound **1** and precursor **2**

Diethyl 2-(((4-bromophenyl)amino)methylene)malonate (**1a**)

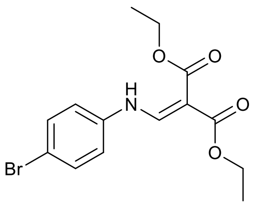

A solution of 4-bromoaniline (15.0 g, 87.2 mmol, 1.00 eq) and diethyl 2-(ethoxymethylene)malonate (20.7 g, 95.9 mmol, 1.10 eq) in EtOH (100 mL) was stirred at 85°C for 4 hours. The reaction was allowed to cool and filtered to isolate diethyl 2-(((4-bromophenyl)amino)methylene)malonate (28.8 g, 96%) as a white crystalline solid.

^{1}H NMR (400 MHz, DMSO-d_6) δ 10.64 (d, *J* = 13.3 Hz, 1H), 8.35 (d, *J* = 13.5 Hz, 1H), 7.55 (d, *J* = 8.9 Hz, 2H), 7.36 (d, *J* = 8.9 Hz, 2H), 4.20 (q, *J* = 7.1 Hz, 2H), 4.12 (q, *J* = 7.1 Hz, 2H), 1.24 (q, *J* = 7.2 Hz, 6H); **LCMS** (ES+) m/z: $[M+H]^+$ 344. Data is in accordance with known literature [1].

Ethyl 6-bromo-4-oxo-1,4-dihydroquinoline-3-carboxylate (**1b**)

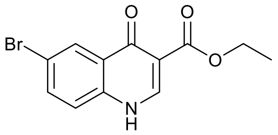

A solution of diethyl 2-(((4-bromophenyl)amino)methylene)malonate (50.5 g, 148 mmol, 1.00 eq) in diphenyl ether (250 mL) was heated to 250°C and stirred for 4 hours open-flask. The reaction was allowed to cool to room temperature and the precipitate was filtered to isolate ethyl 6-bromo-4-oxo-1,4-dihydroquinoline-3-carboxylate (33.5 g, 76.5%) as a beige powder.

^{1}H NMR (400 MHz, DMSO-d_6) δ 8.60 (s, 1H), 8.22 (d, *J* = 2.4 Hz, 1H), 7.76 – 7.69 (m, 1H), 7.58 – 7.51 (m, 1H), 4.19 (q, *J* = 7.0 Hz, 2H), 1.27 (t, *J* = 7.1 Hz, 3H); **LCMS** (ES+) m/z: $[M+H]^+$ 298. Data is in accordance with known literature [1].

6-Bromo-4-oxo-1,4-dihydroquinoline-3-carboxylic acid (**1c**)

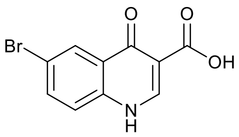

To a stirred solution of ethyl 6-bromo-4-oxo-1,4-dihydroquinoline-3-carboxylate (8.80 g, 29.7 mmol, 1.00 eq) in ethanol (50 mL) was added a solution of NaOH (5.90 g, 149 mmol, 5.00 eq) in H_2O (40 mL) at ambient temperature. The reaction was heated to 80°C and stirred for 2 hours before cooling to 0°C and adjusting to pH 4 with HCl (12 M). The precipitate formed was isolated by filtration affording 6-bromo-4-oxo-1,4-dihydroquinoline-3-carboxylic acid (7.46 g, 93%) as a pink solid.

^{1}H NMR (400 MHz, DMSO-d_6) δ 14.98 (s, 1H), 13.62 (s, 1H), 8.92 (s, 1H), 8.35 (d, *J* = 2.3 Hz, 1H), 8.03 (dd, *J* = 8.9, 2.4 Hz, 1H), 7.79 (d, *J* = 8.9 Hz, 1H); **LCMS** (ES+) m/z: $[M+H]^+$ 270. Data is in accordance with known literature [1].

6-Bromo-4-chloroquinoline-3-carboxamide (**1d**)

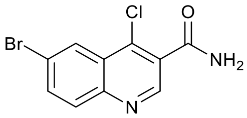

DMF (100 µL) was added to a stirred suspension of 6-bromo-4-oxo-1H-quinoline-3-carboxylic acid (9.49 g, 35.4 mmol, 1.00 eq) in thionyl chloride (50.0 mL, 686 mmol, 19.4 eq) at 0°C and the mixture slowly heated to 75°C reflux, stirring for 2 hours. The reaction was allowed to cool to ambient temperature, concentrated *in vacuo* and the residue dried twice as an azeotrope with anhydrous hexane to afford a yellow solid, which was added portion-wise to saturated aqueous ammonium hydroxide (50 mL) at 0°C. The resulting suspension was stirred for 15 minutes before isolating the solid by filtration and washing with H_2O to afford 6-bromo-4-chloroquinoline-3-carboxamide (9.46 g, 93%) as a beige solid.

^{1}H NMR (400 MHz, DMSO-d_6) δ 8.91 (s, 1H), 8.43 (dd, *J* = 1.9, 0.8 Hz, 1H), 8.23 (s, 1H), 8.08 – 8.05 (m, 2H), 8.01 (s, 1H); **LCMS** (ES+) m/z: $[M+H]^+$ 287. Data is in accordance with known literature [1].

6-Bromo-4-(isopropylamino)quinoline-3-carboxamide (**1e**)

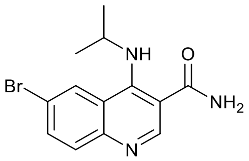

Propan-2-amine (1.03 mL, 12.6 mmol, 1.20 eq) was added to a suspension of 6-bromo-4-chloroquinoline-3-carboxamide (3.00 g, 10.5 mmol, 1.00 eq) and K_2CO_3 (4.35 g, 31.5 mmol, 3.00 eq) in DMF (20 mL) and the mixture stirred at 90°C for 4 hours. The reaction was cooled to ambient temperature and water (100 mL) was added forming a white precipitate that was collected by filtration, washed with pentane and further dried under vacuum to afford 6-bromo-4-(isopropylamino)quinoline-3-carboxamide (3.11 g, 96%) as an off-white powder.

^{1}H NMR (400 MHz, DMSO-d_6) δ 8.60 (s, 1H), 8.48 (d, *J* = 1.8 Hz, 1H), 8.03 (s, 2H), 7.78 (dd, *J* = 8.9, 2.0 Hz, 1H), 7.74 (d, *J* = 8.9 Hz, 1H), 7.50 (s, 1H), 4.13 (s, 1H), 1.23 (d, *J* = 6.3 Hz, 6H); **LCMS** (ES+) m/z: $[M+H]^+$ 310. Data is in accordance with known literature [1].

8-Bromo-1-isopropyl-3H-imidazo[4,5-c]quinolin-2-one (**1f**)

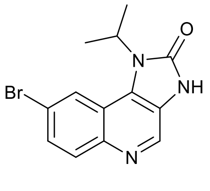

6-Bromo-4-(isopropylamino)quinoline-3-carboxamide (9.18 g, 29.8 mmol, 1.00 eq) and 1,8-diazabicyclo[5.4.0]undec-7-ene (8.90 mL, 59.6 mmol, 2.00 eq) were suspended in MeOH (61.2 mL) and cooled to 0°C before adding a solution of trichloroisocyanuric acid (3.46 g, 14.9 mmol, 0.50 eq) in methanol (20 mL) dropwise over 10 minutes. The reaction was returned to ambient temperature and stirred for 20 hours before filtering, isolating 8-bromo-1-isopropyl-3H-imidazo[4,5-c]quinolin-2-one (5.08 g) as a cream solid. Further material was isolated by concentrating the filtrate and resuspending in water (50 mL) before adjusting to pH 3 (HCl, 1 M) and filtering the resulting precipitate (3.51 g). Overall yield 8.59 g, 94.2%.

^{1}**H NMR** (400 MHz, DMSO-d_6) δ 11.54 (s, 1H), 8.66 (s, 1H), 8.40 (d, *J* = 1.9 Hz, 1H), 7.95 (d, *J* = 9.0 Hz, 1H), 7.72 (dd, *J* = 9.0, 2.0 Hz, 1H), 5.12 (hept, *J* = 6.7 Hz, 1H), 1.62 (d, *J* = 6.8 Hz, 6H); **LCMS** (ES+) m/z: $[M+H]^+$ 308. Data is in accordance with known literature [1].

8-Bromo-1-isopropyl-3-methyl-1,3-dihydro-2H-imidazo[4,5-c]quinolin-2-one (**1g**)

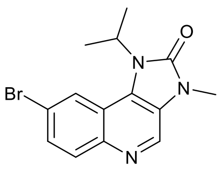

8-Bromo-1-isopropyl-1,3-dihydro-2H-imidazo[4,5-c]quinolin-2-one (7.80 g, 25.5 mmol, 1.00 eq), tetrabutylammonium bromide (821 mg, 2.55 mmol, 0.100 eq) and methyl iodide (3.20 mL, 51.0 mmol, 2.00 eq) were suspended in DCM (100 mL) before adding a solution of NaOH (1.53 g, 38.2 mmol, 1.50 eq) in water (50 mL) and stirring vigorously at ambient temperature for 3 hours. The reaction was concentrated removing DCM *in vacuo* before diluting with MeOH (30 mL) and filtering to isolate 8-

bromo-1-isopropyl-3-methyl-1,3-dihydro-2H-imidazo[4,5-c]quinolin-2-one (6.86 g, 84%) as a white powder.

^{1}H NMR (400 MHz, DMSO-d_6) δ 8.92 (s, 1H), 8.44 (s, 1H), 7.99 (d, *J* = 9.1 Hz, 1H), 7.75 (dd, *J* = 9.2, 2.0 Hz, 1H), 5.19 (hept, *J* = 6.8 Hz, 1H), 3.49 (s, 3H), 1.63 (d, *J* = 6.7 Hz, 6H); **LCMS** (ES+) m/z: $[M+H]^+$ 322. Data is in accordance with known literature [1].

1-isopropyl-3-methyl-8-(4,4,5,5-tetramethyl-1,3,2-dioxaborolan-2-yl)-1,3-dihydro-2H-imidazo[4,5-c]quinolin-2-one (**1h**)

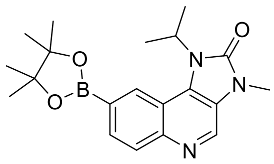

In a flame-dried two-necked 25 mL round bottom flask, 8-bromo-1-isopropyl-3-methyl-1,3-dihydro-2*H*-imidazo[4,5-*c*]quinolin-2-one (500 mg, 1.56 mmol, 1.00 eq), potassium acetate (460 mg, 4.68 mmol, 3.00 eq) and bis(pinacolate)diboron (594 mg, 2.34 mmol, 1.50 equiv) were dissolved in anhydrous 1,4-dioxane (25 mL) under an argon atmosphere. The mixture was stirred and flushed with argon for 15 minutes before adding $Pd(dppf)Cl_2 \cdot CHCl_2$ (64.0 mg, 5% mol) and flushing for a further 15 minutes. The reaction was heated to 80°C, stirred for 24 hours, then allowed to cool to room temperature and filtered through Celite (3 cm) eluting with EtOAc (500 mL). The filtrate was reduced to a fifth of its volume before partitioning with H_2O (100 mL) and the aqueous phase re-extracted with further EtOAc (100 mL). The combined organics were washed with brine (100 mL), dried over $MgSO_4$ and concentrated *in vacuo* to a crude brown solid. The solid was suspended in *n*-pentane (40 mL), sonicated and filtered to afford 1-isopropyl-3-methyl-8-(4,4,5,5-tetramethyl-1,3,2-dioxaborolan-2-yl)-1,3-dihydro-2*H*-imidazo[4,5-*c*]quinolin-2-one (470 mg, 82%) as a brown powder, essentially pure according to ^{1}H NMR.

^{1}H NMR (400 MHz, $CDCl_3$) δ 8.73–8.66 (m, 2H), 8.10 (d, *J* = 8.4 Hz, 1H), 7.94 (dd, *J* = 8.5, 1.3 Hz, 1H), 5.31 (p, *J* = 6.9 Hz, 1H), 3.55 (s, 3H), 1.78 (d, *J* = 7.0 Hz, 6H), 1.38 (s, 12H). **^{13}C NMR** (101 MHz, $CDCl_3$) δ 153.7, 146.8, 133.0, 131.4, 130.1, 129.6, 122.9, 115.28, 84.2, 67.1, 27.2, 24.9, 20.5. **HRMS** (ESI) for $C_{20}H_{26}BN_3O_3$ $[M+H]^+$ requires 368.2141 found 368.2141.

5-Bromo-6-fluoropyridin-2-amine (**1i**)

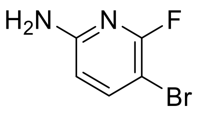

N-bromosuccinamide (16.7 g, 93.7 mmol, 1.05 eq) was added portion-wise over 25 minutes to a solution of 6-fluoropyridin-2-amine (10.0 g, 89.2 mmol, 1.00 eq) in acetonitrile (80 mL) maintained at 10-20°C. The reaction was stirred at ambient temperature for 1.5 hours then concentrated *in vacuo* to a brown residue which was triturated in water (100 mL) and the solid filtered to isolate 5-bromo-6-fluoropyridin-2-amine (15.1 g, 89%) as a pale brown solid.

^{1}H NMR (400 MHz, $CDCl_3$) δ 7.59 (dd, *J* = 8.9, 8.3 Hz, 1H), 6.25 (dd, *J* = 8.3, 1.4 Hz, 1H), 3.84 (s, 2H). **LCMS** (ES) m/z: $[M + H]^+$ 192. Data is in accordance with known literature [1].

5-Bromo-6-fluoropyridin-2-ol (**1j**)

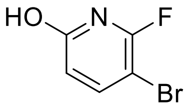

5-Bromo-6-fluoropyridin-2-amine (4.00 g, 20.9 mmol, 1.00 eq) was suspended in a solution of H_2SO_4 (18 M, 11.6 mL, 209 mmol, 10.0 eq) in H_2O (30 mL) and cooled to 0°C before adding a solution of $NaNO_2$ (4.34 g, 62.8 mmol, 3.00 eq) in water (10 mL) dropwise over 1 hour, maintaining the temperature below 5°C. The reaction was then stirred at 0°C for 1 hour before allowing it to warm to room temperature and stirring overnight for a total of 24 hours. The precipitate was then collected by filtration to isolate 5-bromo-6-fluoropyridin-2-ol (3.30 g, 82%) as a brown solid.

^{1}H NMR (400 MHz, $CDCl_3$) δ 10.16 (s, 1H), 7.85 (t, *J* = 8.7 Hz, 1H), 6.59 (dd, *J* = 8.5, 0.7 Hz, 1H); **LCMS** (ES) m/z: $[M + H]^+$ 192. Data is in accordance with known literature [1].

3-Bromo-2-fluoro-6-(3-(piperidin-1-yl)propoxy)pyridine (**1k**)

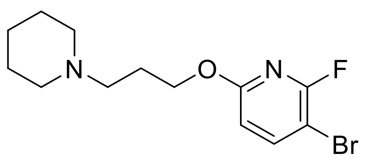

5-Bromo-6-fluoropyridin-2-ol (2.62 g, 13.6 mmol, 1.00 eq) was dissolved in DMF (80 mL), then 1-(3-chloropropyl)piperidine monohydrochloride (3.35 g, 16.4 mmol, 1.20 eq) and potassium carbonate (5.66 g, 40.9 mmol, 3.00 eq) were added and the mixture stirred at 100°C for 24 hours. The reaction was cooled to room temperature before adding water (100 mL) and extracting with ethyl acetate (4 × 30 mL). Combined organics washed with aqueous LiCl (1 M, 100 mL) then brine (100 mL), dried over $MgSO_4$ and concentrated to a red oil. The extracted crude was purified by silica gel chromatography, eluting with 0-2% 2N methanolic ammonia in ethyl acetate to isolate 3-bromo-2-fluoro-6-(3-(piperidin-1-yl)propoxy)pyridine (2.71 g, 63%) as a red oil.

^{1}H NMR (400 MHz, $CDCl_3$) δ 7.73 (t, *J* = 8.8 Hz, 1H), 6.51 (dd, *J* = 8.4, 0.9 Hz, 1H), 4.27 (t, *J* = 6.5 Hz, 2H), 2.45–2.30 (m, 6H), 1.99–1.88 (m, 2H), 1.57 (p, *J* = 5.6 Hz, 6H), 1.47–1.37 (m, 2H). **^{13}C NMR** (101 MHz, $CDCl_3$) δ 162.2, 159.2, 156.8, 145.3, 109.3, 93.3, 92.9, 77.4, 66.0, 56.0, 54.8, 26.5, 26.1, 24.6. **^{19}F NMR** (376 MHz, $CDCl_3$) δ -67.11 (d, *J* = 9.2 Hz). **HRMS** (ESI) for $C_{13}H_{18}BrFN_2O$ $[M+H]^+$ requires 319.0639, found 319.0634. Data is in accordance with known literature [1].

8-(2-fluoro-6-(3-(piperidin-1-yl)propoxy)pyridin-3-yl)-1-isopropyl-3-methyl-1,3-dihydro-2H-imidazo[4,5-c]quinolin-2-one (**1**)

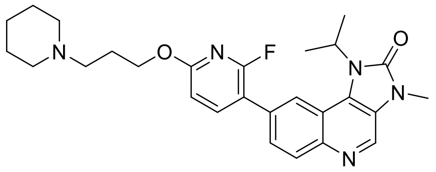

In a two neck 25 mL round bottom flask, 3-bromo-2-fluoro-6-(3-(piperidin-1-yl)propoxy)pyridine (104 mg, 0.328 mmol, 1.00 eq), was added and dissolved in 1,4-dioxane (3.60 mL) and H_2O (1.80 mL). 1-Isopropyl-3-methyl-8-(4,4,5,5-tetramethyl-1,3,2-dioxaborolan-2-yl)-1,3-dihydro-2H-imidazo[4,5-c]quinolin-2-one (132 mg, 0.361 mmol, 1.10 eq) and K_2CO_3 (136 mg, 0.984 mmol, 3.00 eq) were added and flushed with argon for 5 min before adding tetrakis(triphenylphosphine)palladium(0) (19.0 mg, 0.016 mmol, 0.05 eq) and heating to 100°C reflux for 24 hours. The reaction was allowed to cool before passing through Celite, eluting with acetone (150 mL) and drying to a brown crude oil. The crude was suspended in water and extracted with EtOAc (3 x 50 mL), combined organics were washed with brine (100 mL), dried over $MgSO_4$ and concentrated *in vacuo* to a yellow residue (110 mg). The extracted crude was purified by silica gel chromatography, first eluting impurities with methanol, then the product was eluted in 4% 2N methanolic ammonia in DCM, isolating 8-(2-fluoro-6-(3-(piperidin-1-yl)propoxy)pyridin-3-yl)-1-isopropyl-3-methyl-1,3-dihydro-2H-imidazo[4,5-c]quinolin-2-one (51 mg, 33%) as a white crystalline solid.

^{1}H NMR (400 MHz, $CDCl_3$) δ 8.69 (s, 1H), 8.43 (s, 1H), 8.19 (d, *J* = 8.8 Hz, 1H), 7.90 (dd, *J* = 10.2, 8.2 Hz, 1H), 7.72 (d, *J* = 8.9 Hz, 1H), 6.77 (dd, *J* = 8.2, 1.0 Hz, 1H), 5.26 (p, *J* = 7.0 Hz, 1H), 4.38 (t, *J* = 6.5 Hz, 2H), 3.58 (s, 3H), 2.52–2.44 (m, 2H), 2.41 (s, 4H), 2.06–1.99 (m, 2H), 1.76 (d, *J* = 6.9 Hz, 6H), 1.59 (p, *J* = 5.6 Hz, 4H), 1.44 (q, *J* = 6.0 Hz, 2H). **^{13}C NMR** (101 MHz, $CDCl_3$) δ 162.34 (d, *J* = 13.9 Hz), 159.9, 157.5, 153.7, 144.5, 142.8 (d, *J* = 4.3 Hz), 132.6, 131.8 (d, *J* = 5.4 Hz), 131.6, 129.3, 126.8 (d, *J* = 1.8 Hz), 123.3, 115.6, 113.7 (d, *J* = 26.3 Hz), 108.4 (d, *J* = 5.2 Hz), 65.6, 55.9, 54.6, 27.3, 26.3, 25.7, 24.3, 20.5. **^{19}F NMR** (376

MHz, $CDCl_3$) δ -73.3 (d, *J* = 10.1 Hz) (potential rotamer). **HRMS** (ESI) for $C_{27}H_{32}FN_5O_2$ $[M+H]^+$ requires 478.2613, found 478.2610. Data is in accordance with known literature [1].

5-Bromo-6-nitropyridin-2-amine (**2a**)

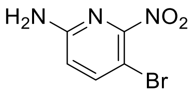

A solution of 6-nitropyridin-2-amine (3.03 g, 21.7 mmol, 1.00 eq) in acetonitrile (200 mL) was cooled to 0°C and protected from light before adding *N*-bromosuccinamide (3.87 g, 21.7 mmol, 1.00 eqv) portion-wise over 1.5 h. The reaction was allowed to reach room temperature overnight before addition of saturated aqueous $NaHCO_3$ (50 mL) and H_2O (100 mL) and extraction into ethyl acetate (3 x 100 mL). The combined organic fractions were washed with brine (100 mL), dried over magnesium sulphate, and concentrated *in vacuo* to afford a yellow solid. The product was purified by silica gel chromatography eluting with 10-50% ethyl acetate in *n*-pentane, affording pure 5-bromo-6-nitropyridin-2-amine (1.20 g, 25%).

^{1}HNMR (400 MHz, DMSO) δ 7.84 (d, *J* = 8.8 Hz, 1H), 7.01 (s, 2H), 6.66 (d, *J* = 8.8 Hz, 1H). **HRMS** (ESI) for $C_5H_4BrN_3O_2$ $[M+H]^+$ requires 218.9223 found 218.9224. Data is in accordance with known literature [2].

5-Bromo-6-nitropyridin-2-ol (**2b**)

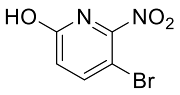

A solution of 5-Bromo-6-nitropyridin-2-amine (2.11 g, 9.69 mmol, 1.00 eqv) in H_2O (150 mL) was cooled to 0°C before addition of H_2SO_4 (18 M, 5.40 mL, 97.20 mmol, 10.0 eqv). A cooled solution of

$NaNO_2$ (2.01 g, 29.1 mmol, 3.00 eqv) in water (25 mL) was then added portion-wise. The resulting suspension was stirred vigorously for at 0°C for 45 min, then allowed to return to room temperature, stirring overnight. The precipitate was then collected by filtration and dried with pentane to isolate 5-Bromo-6-nitropyridin-2-ol (1.79 g, 85%) as a brown solid.

^{1}H NMR (400 MHz, Acetone) δ 10.83 (s, 1H), 8.20 (d, *J* = 8.6 Hz, 1H), 7.05 (d, *J* = 8.6 Hz, 1H). **^{13}C NMR** (101 MHz, Acetone) δ 162.9, 156.19, 147.6, 116.4, 99.1. **HRMS** (ESI) for $C_5H_3BrN_2O_3$ $[M-H]^-$ requires 218.9223, found 218.9224.

3-Bromo-2-nitro-6-(3-(piperidin-1-yl)propoxy)pyridine (**2c**)

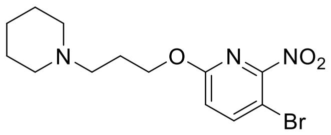

5-Bromo-6-nitropyridin-2-ol (100 mg, 0.457 mmol, 1.00 eq) was dissolved in DMF (8 mL), then 1-(3-chloropropyl)piperidine monohydrochloride (112 mg, 0.548 mmol, 1.20 eq) and K_2CO_3 (189 mg, 1.37 mmol, 3.00 eq) were added and the mixture stirred at 80°C for 24 hours. The reaction was cooled to room temperature before adding H_2O (8 mL) and extracting with ethyl acetate (4 × 7 mL). Combined organics washed with aqueous LiCl (1 M, 20 mL) then brine (20 mL), dried over $MgSO_4$ and concentrated to a brown oil. The extracted crude was purified by silica gel chromatography, eluting with 2-10% methanol in dichloromethane to isolate 3-bromo-2-nitro-6-(3-(piperidin-1-yl)propoxy)pyridine (98.0 mg, 62%) as a brown oil.

^{1}H NMR (400 MHz, $CDCl_3$) δ 7.86 (d, *J* = 8.7 Hz, 1H), 6.83 (d, *J* = 8.7 Hz, 1H), 4.31 (t, *J* = 6.5 Hz, 2H), 2.46 – 2.32 (m, 6H), 2.00 – 1.88 (m, 2H), 1.56 (p, *J* = 5.6 Hz, 4H), 1.41 (p, *J* = 6.1 Hz, 2H). **^{13}C NMR** (101 MHz, $CDCl_3$) δ 207.0, 161.9, 145.6, 116.6, 100.2, 77.4, 66.4, 55.7, 54.6, 31.0, 26.1, 25.9, 24.4 . **HRMS** (ESI) for $C_{13}H_{18}BrN_3O_3$ $[M+H]^+$ requires 346.0595, found 346.0579.

1-Isopropyl-3-methyl-8-(2-nitro-6-(3-(piperidin-1-yl)propoxy)pyridin-3-yl)-1,3-dihydro-2H-imidazo[4,5-c]quinolin-2-one (**2**)

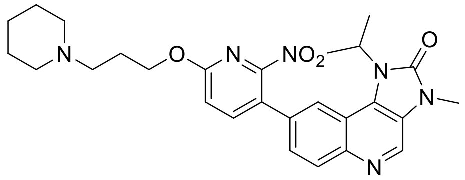

In a two neck 25 mL round bottom flask, 3-bromo-2-nitro-6-(3-(piperidin-1-yl)propoxy)pyridine (248 mg, 0.720 mmol, 1.00 eq), was added and dissolved in 1,4-dioxane (11.7 mL) and H_2O (1.3 mL). 1-Isopropyl-3-methyl-8-(4,4,5,5-tetramethyl-1,3,2-dioxaborolan-2-yl)-1,3-dihydro-2H-imidazo[4,5-c]quinolin-2-one (529 mg, 1.44 mmol, 2.00 eq) and caesium carbonate (704 mg, 2.16 mmol, 3.00 eq) were added and flushed with argon for 10 min before adding tetrakis(triphenylphosphine)palladium(0) (250 mg, 0.216 mmol, 0.300 eq), flushed with argon for a further 10 min, then heated to 90°C reflux for 15 hours. The reaction was allowed to cool before passing though celite eluting with ethyl acetate (50 mL), the collected organic washed with water (60 mL) and brine (60 mL), dried over $MgSO_4$ and concentrated *in vacuo* to a yellow residue (610 mg). The extracted crude was purified by silica gel chromatography eluting with 0-20% methanol in dichloromethane, isolating 1-isopropyl-3-methyl-8-(2-nitro-6-(3-(piperidin-1-yl)propoxy)pyridin-3-yl)-1,3-dihydro-2H-imidazo[4,5-c]quinolin-2-one (102 mg, 28%) as a yellow crystalline solid.

^{1}H NMR (400 MHz, $CDCl_3$) δ 8.72 (s, 1H), 8.21 (d, *J* = 8.8 Hz, 1H), 8.12 (d, *J* = 1.9 Hz, 1H), 7.87 (d, *J* = 8.4 Hz, 1H), 7.54 (dd, *J* = 8.7, 1.9 Hz, 1H), 7.09 (d, *J* = 8.5 Hz, 1H), 5.19 – 5.04 (m, 1H), 4.45 (t, *J* = 6.4 Hz, 2H), 3.57 (s, 3H), 2.62 – 2.40 (m, 6H), 2.07 (p, *J* = 6.6 Hz, 2H), 1.73 (d, *J* = 6.9 Hz, 6H), 1.66 (p, *J* = 5.7 Hz, 4H), 1.51 – 1.43 (m, 4H). **^{13}C NMR** (101 MHz, $CDCl_3$) δ 162.3, 155.1, 153.6, 144.9, 143.3, 133.1, 132.3, 132.3, 129.3, 126.5, 123.6, 121.4, 115.6, 115.4, 66.0, 55.7, 54.5, 31.0, 29.8, 27.4, 25.9, 25.5, 24.1, 20.6. **HRMS** (ESI) for $C_{27}H_{32}N_6O_4$ $[M+H]^+$ requires 505.2558, found 505.2557.

2. Automation of radiosynthesis

Automation of the radiosynthesis of [^{18}F]**1** was performed on an Eckert & Ziegler Modular-lab system (figure S2). Briefly, [^{18}F]fluoride (5-10 GBq, supplied by PETIC, Cardiff or Alliance, UK) was trapped on a QMA carbonate cartridge (Waters, preconditioned with 2 mL deionised H_2O) and eluted to the reaction vessel with a solution of Kryptofix®222 (30 mg) and K_2CO_3 (6 mg) in H_2O (0.1 mL) and acetonitrile (0.9 mL), driven by vacuum. The [^{18}F]fluoride was azeotropically dried with three further additions of anhydrous acetonitrile (3 × 0.5 mL) at 120°C under vacuum and nitrogen air flow, before addition of precursor **2** (1 mg) in DMF (300 µL) and reaction at 120°C for 10 min. The reaction was allowed to cool to 35°C then diluted by addition of 0.1% formic acid in water (4.5 mL) before loading onto a Luna 10u C18(2) 100 Å 250 x 10 mm semi-preparative column. The product was eluted with 25% acetonitrile in 0.1% formic acid buffer (isocratic) at a flow rate of 3 mL/min. The product peak was redirected and diluted in a flask containing dH_2O (15 mL). The diluted product was then driven with nitrogen and trapped on a C18 light cartridge (Waters, preconditioned with 10 mL EtOH then 10 mL dH_2O). The C18 cartridge was washed with dH_2O (1 mL) before eluting to the final vial with ethanol absolute (1.5 mL) which was then dried at 100°C under vacuum until dry. The dried product was reformulated in a solution of 10% DMSO/0.9% saline (typically at 0.5-1 GBq/mL) and 20 µL sample used for quality control performed on a ThermoScientific UltiMate™ 3000 system with a Luna Omega 5µm Polar C18 100 Å LC 250 x 4.6 mm analytical column, eluting with 25% acetonitrile in 0.1% formic acid buffer (isocratic) at a flow rate of 1 mL/min. A secondary sample (10 µL) was co-injected with a solution of reference [^{19}F]**1** to confirm product identity.

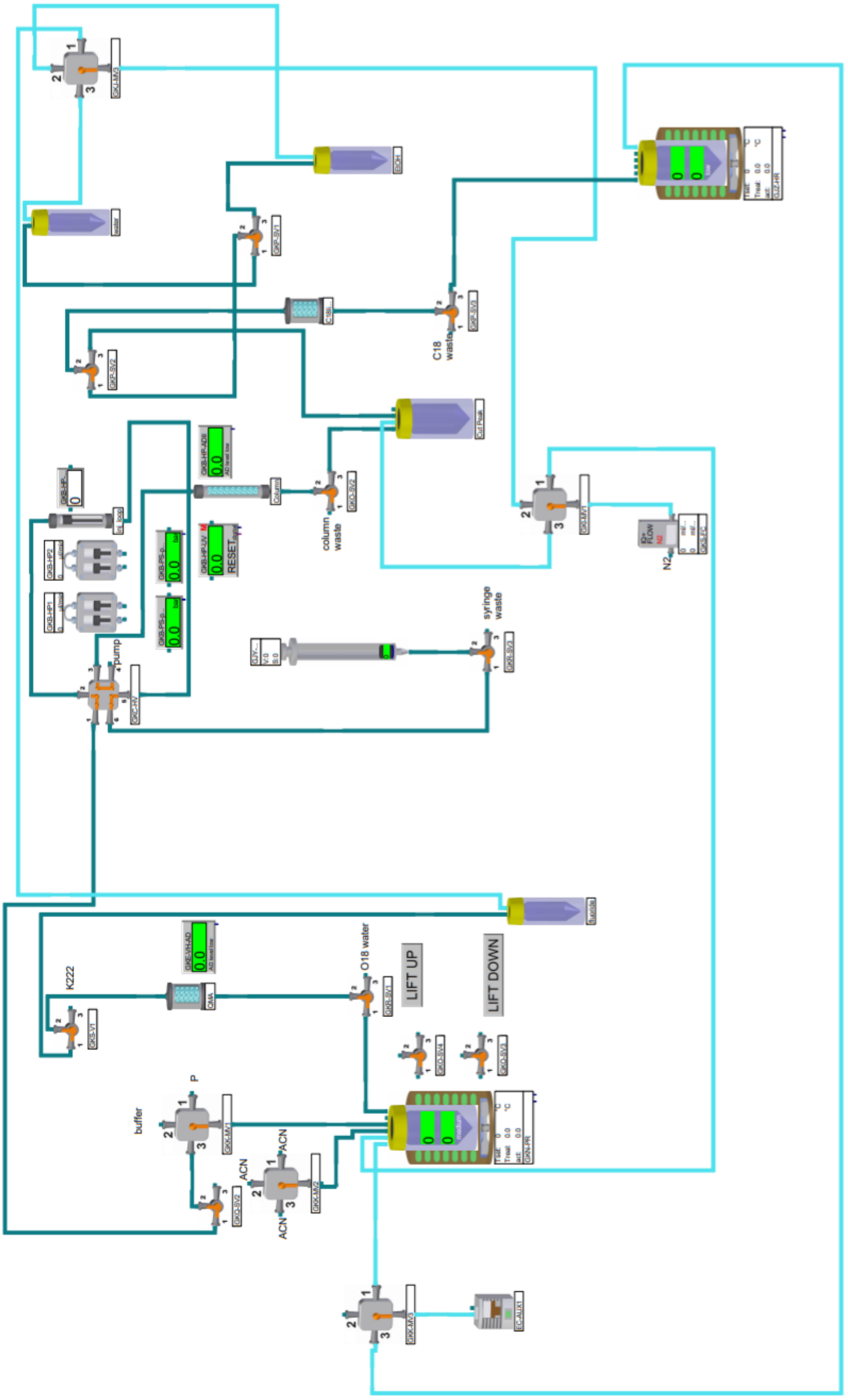

Supplementary Fig. S2 Eckert & Ziegler Modular-Lab setup for automated radiosynthesis of [^{18}F]**1**.

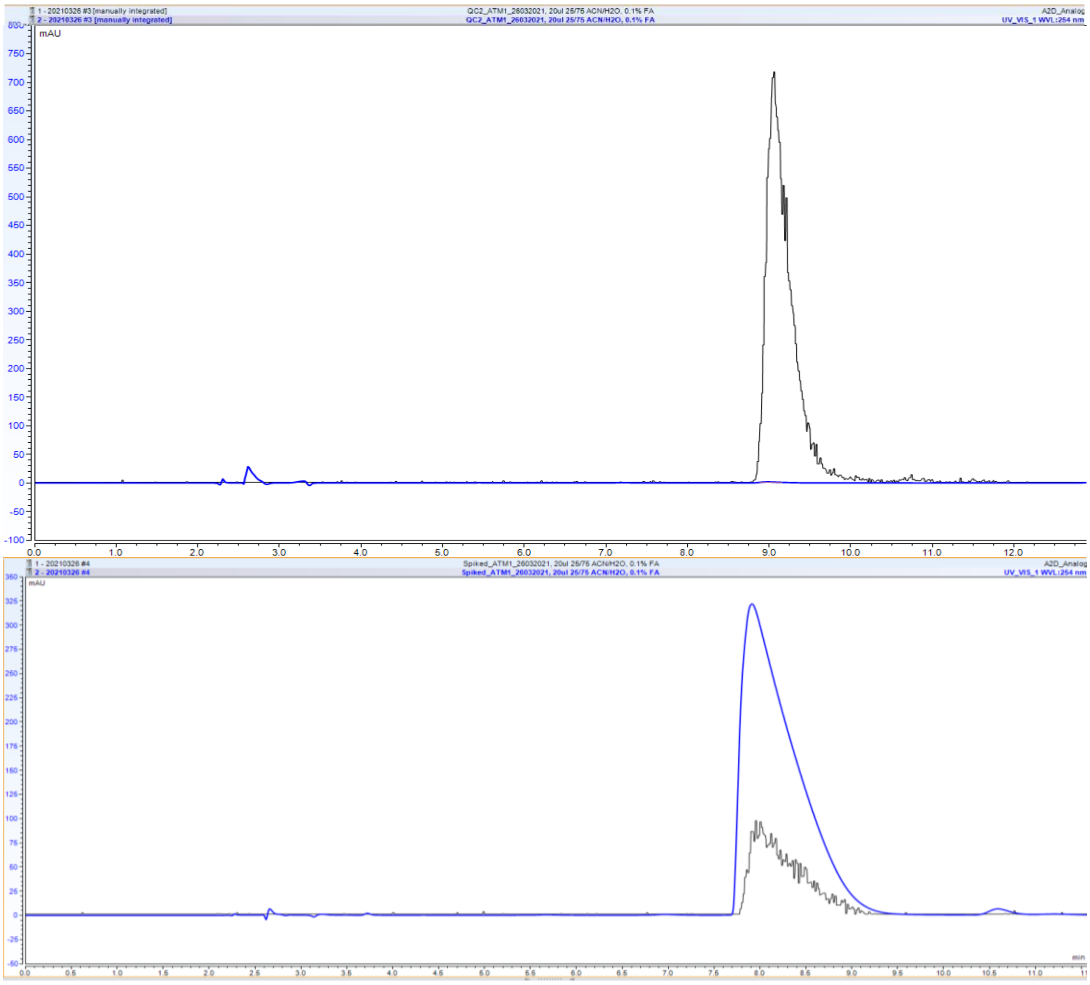

Supplementary Fig. S3 Example quality control trace of [^{18}F]**1**. Top trace shows reformulated [^{18}F]**1**, bottom trace shows co-injection of reformulated [^{18}F]**1** with reference [^{19}F]1. Radio trace in black, UV trace (254 nm) in blue.

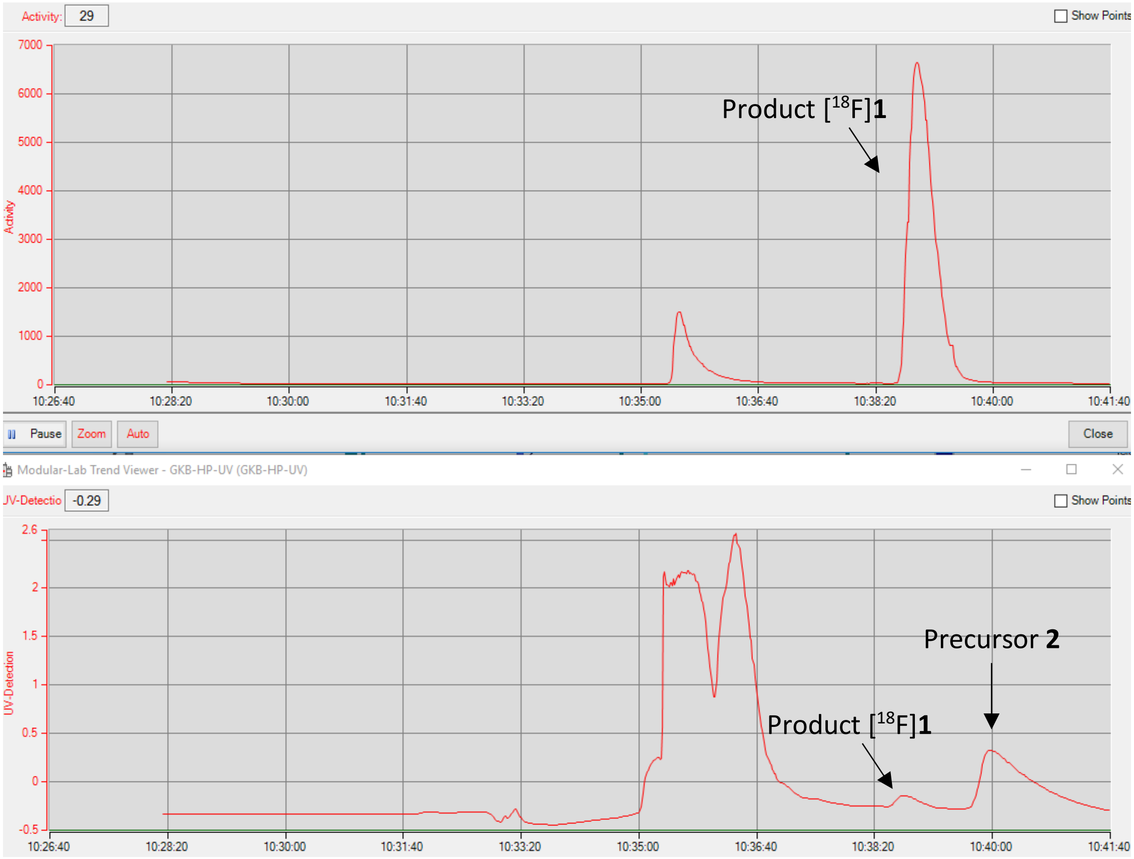

Supplementary Fig. S4 Example semi-preparatory trace of [^{18}F]**1** (radio trace top, UV trace (220 nm) bottom).

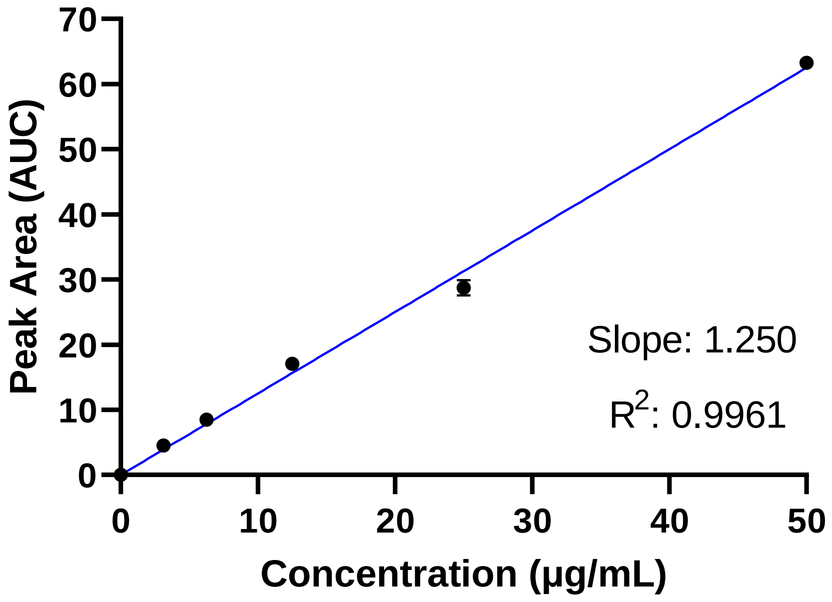

Supplementary Fig. S5 Calibration curve used for molar activity calculations of **1**

3. Supplementary Figures

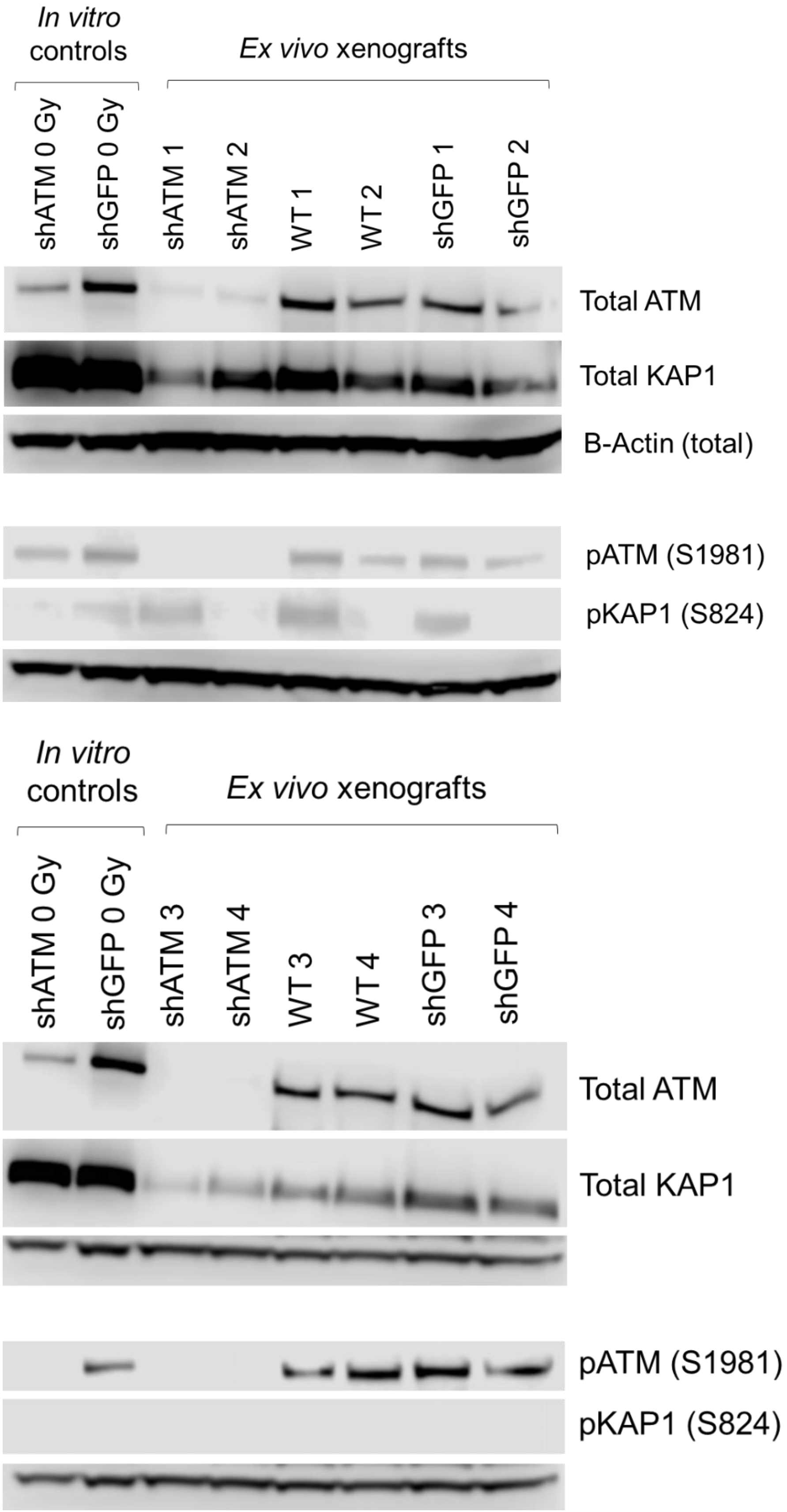

Supplementary Fig. S6 Representative western blot of *ex vivo* xenograft samples

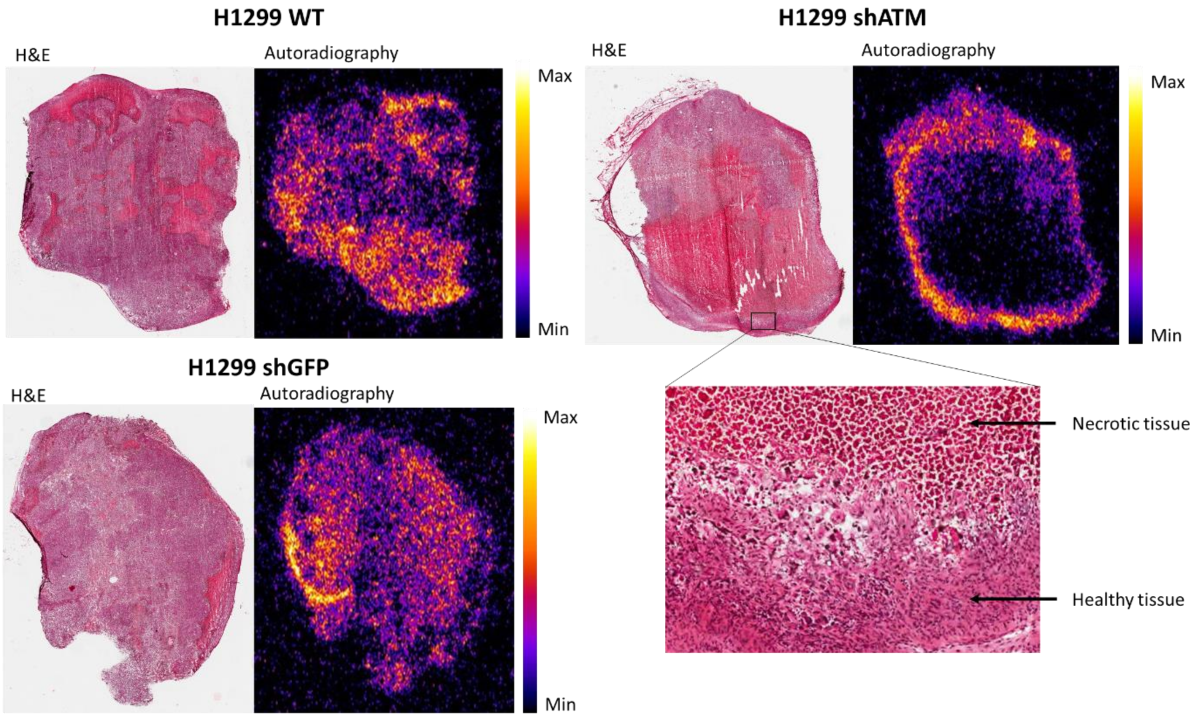

Supplementary Fig. S7 Autoradiography and H&E staining of xenograft sections (10 µm)

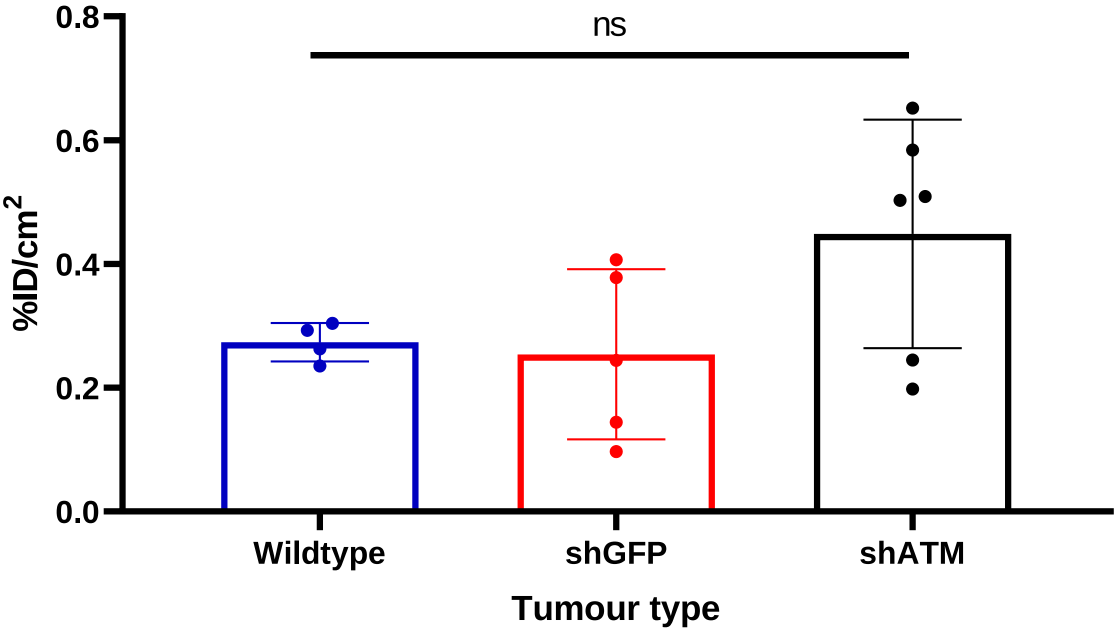

Supplementary Fig. S8 Autoradiography signal analysis of *ex vivo* tumour sections.

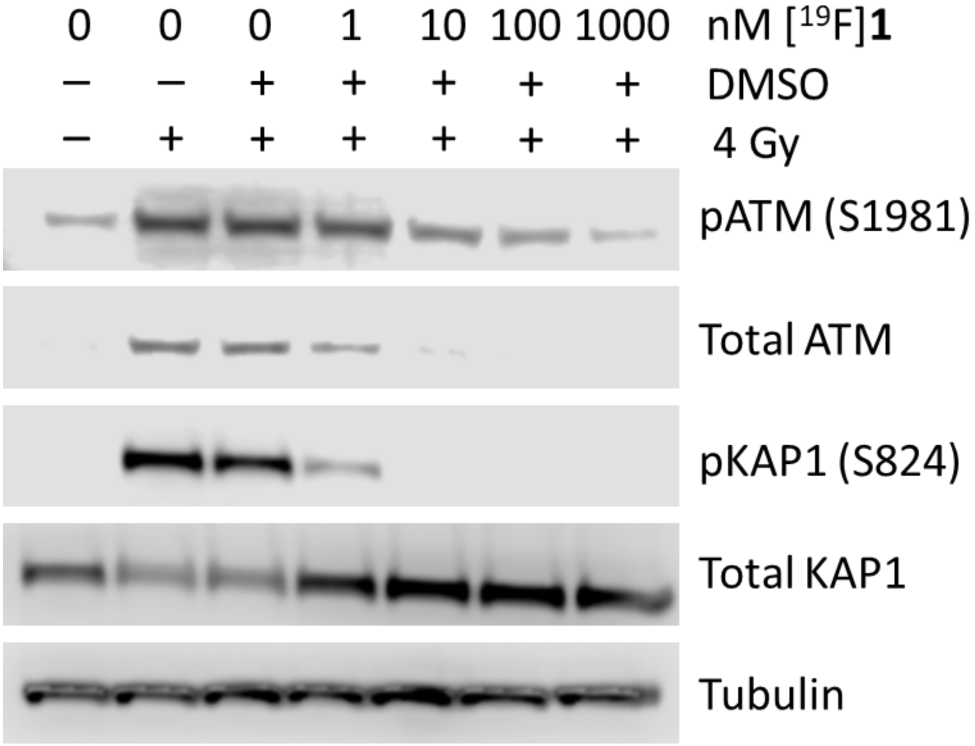

Supplementary Fig. S9 ATM inhibition with [^{19}F]**1** in CT26 murine colorectal cancer cells. Antibodies used are stated in the materials and methods, with a replacement of the total ATM Abcam antibody with GTX70107, 1:500 (GeneTex).

4. Western Blot full protocol

After the required treatments, cell culture medium was removed and cells were washed once with ice cold PBS. Cells were then lysed in RIPA buffer containing protease inhibitor (cOmplete EDTA-free, Roche) and phosphatase inhibitor (PhosStop, Roche) and collected by scraping. Lysates were kept on ice with agitation for 20 min then centrifuged at 20,000 rcf, 4°C for 20 minutes and supernatant (lysate) isolated.

For xenograft lysis, the xenografts were weighed and supplemented RIPA buffer added (20 µL/mg sample) immediately following thaw. Tissue was mechanically broken up using a scalpel and by passing though 19G and 21G needles. The lysates were agitated via vortex and sonicated (3 x 10 s) periodically while keeping on ice for 20 min before being centrifuged at 20,000 rcf, 4°C for 20 minutes and supernatant (lysate) isolated.

Lysates were normalised against BSA standards via BCA assay before reduction and denaturation in NuPage LDS sample buffer (Invitrogen) containing DTT at 100 mM final concentration for 10 min at 70°C. Samples were run at ~30 mg per well on NuPage® 3-8% Tris-Acetate gel in Tris-acetate Running Buffer (both Novex) at 150 V. Gels were blotted onto PVDF membranes using the iBlot2 system (Invitrogen) (20 V for 1 min, 23 V for 3 min, 25 V for 5 min) and recovered into tris-buffered saline (TBS, Santa Cruiz).

Blots were then probed with a variety of antibodies using the following sequence: Blots were blocked for 1 hour at room temperature in 5% milk/TBST (TBS with 0.1% Tween-20) solution, then submerged in a solution of primary antibody at the required dilution in block solution and incubated at 4°C overnight. Blots were then washed 3 × 15 min in TBST. If required, blots were then incubated for 1 hour at room temperature in a solution of HRP-conjugated secondary antibody in block solution, then washed 3 × 15 min in TBST. Blots were rinsed in TBS, then incubated at room temperature using a SuperSignal™ West Pico PLUS chemiluminescence kit (Thermo Scientific) for 5 min before imaging on a Li-Cor C-DiGit Blot Scanner. After imaging, blots were recovered in TBS and washed 3 × 15 min in

TBST before blocking, repeating the same sequence with additional primary antibodies. Separate identical blots were used to probe for phosphorylated proteins and their total protein counterparts, with protein standards stained for on each blot to compare loading. Blots were cut along ~170 kDa to separate ATM bands, allowing for separate, simultaneous staining.

5. H&E procedure

Frozen tissue section slides were allowed to air dry for 10 min at room temperature before washing in PBS (2 x 3 min) to remove any residual OCT. Slides were then submerged in 4% formaldehyde in PBS for 10 min to fix. Slides were washed in deionised water (3 x 2 min) before submerging in 0.1% Mayers Haematoxylin Solution (Sigma Aldrich) for 8 min at room temperature. Slides were then rinsed in cool running tap water for 6 min followed by rinsing in deionised water (2 x 2 min). Slides were dipped in Eosin Y-Solution 0.5% alcoholic (Merck) for 3.5 min then washed in distilled water for 2 min. Slides were dehydrated by dipping in ethanol solutions (70%, 90%, then 100%, 30 sec each) and cleared in xylene (30 sec) before mount and coverslip with DPX. Mounted slides were allowed to dry overnight before imaging at 20x on a Leica Aperio slide scanner.

6. ^{1}H, ^{13}C and ^{19}F NMR Spectra of Novel Compounds

1-isopropyl-3-methyl-8-(4,4,5,5-tetramethyl-1,3,2-dioxaborolan-2-yl)-1,3-dihydro-2H-imidazo[4,5-c]quinolin-2-one

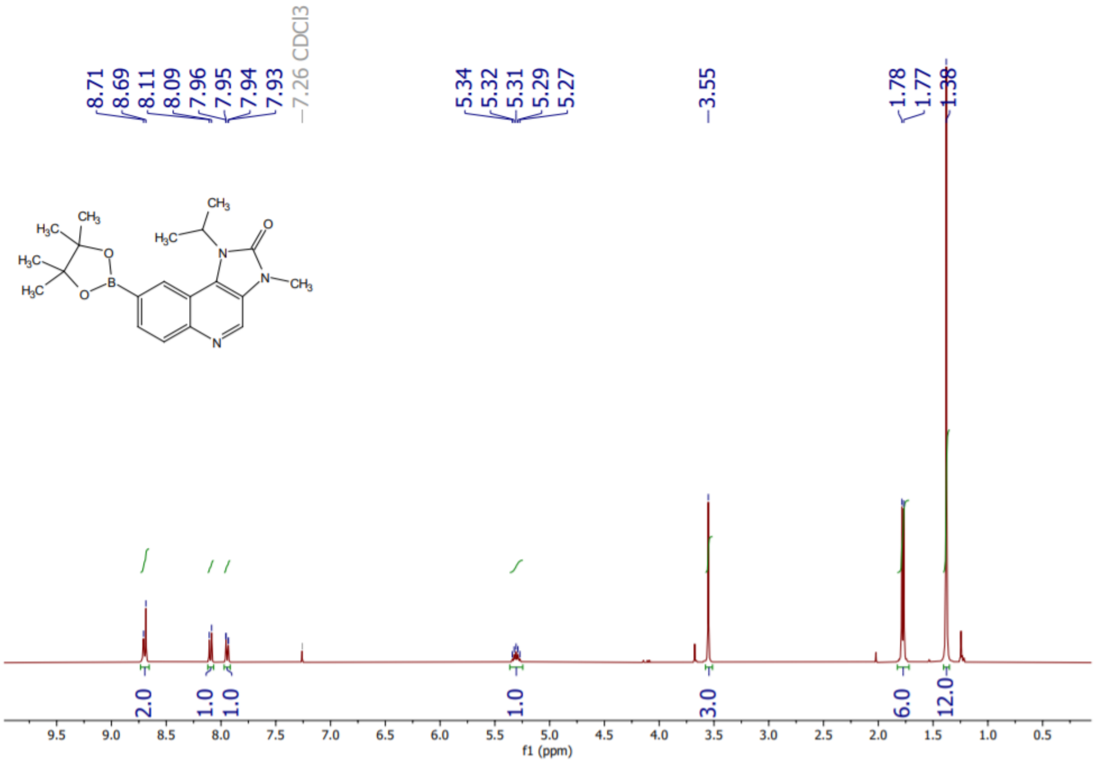

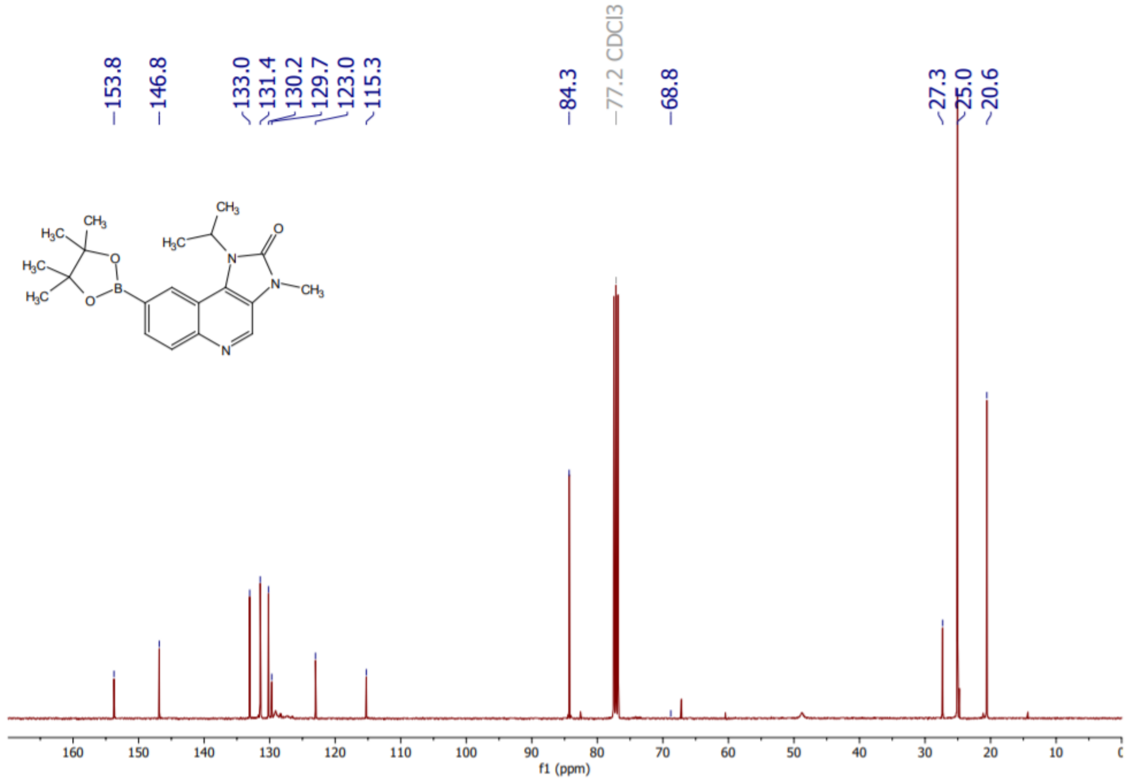

8-(2-fluoro-6-(3-(piperidin-1-yl)propoxy)pyridin-3-yl)-1-isopropyl-3-methyl-1,3-dihydro-2H-imidazo[4,5-c]quinolin-2-one

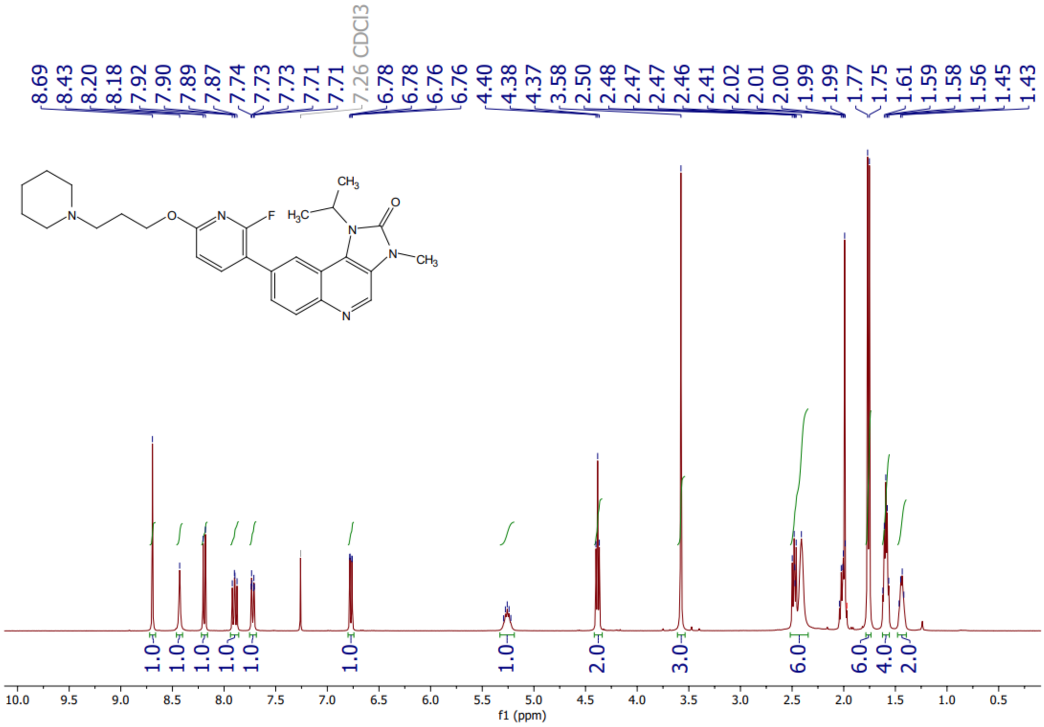

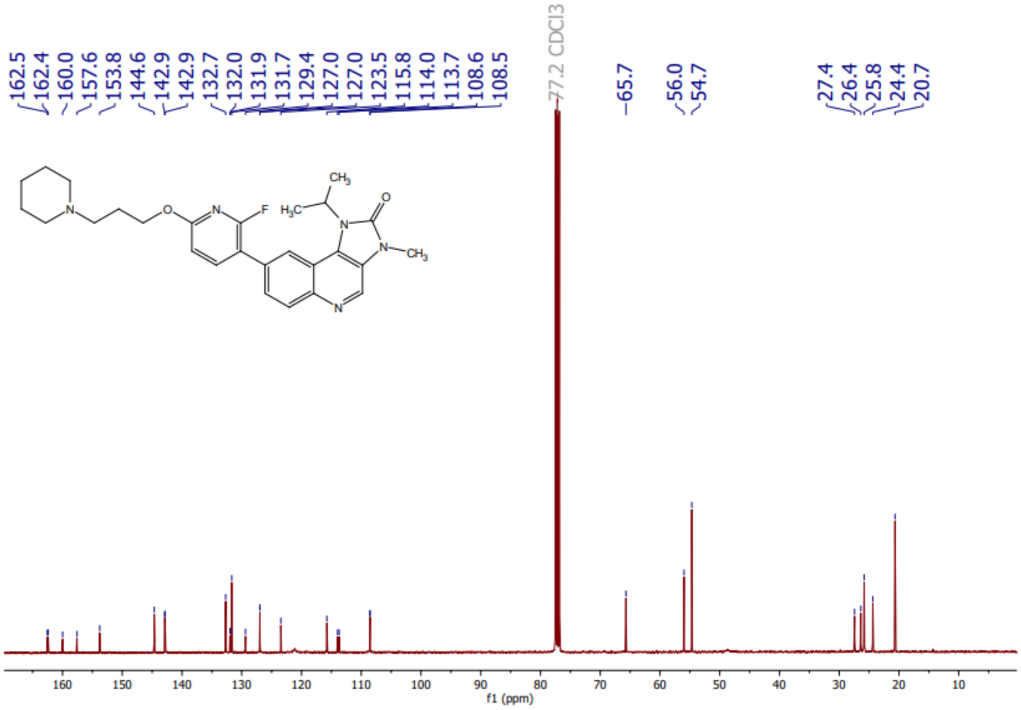

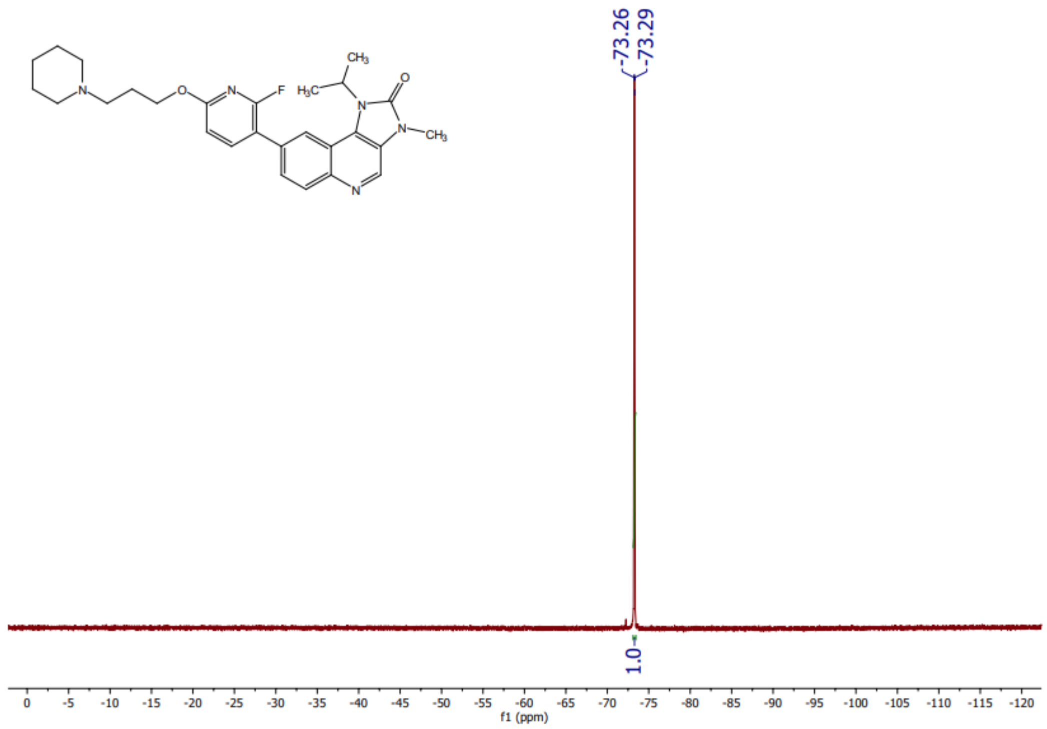

5-Bromo-6-nitropyridin-2-ol

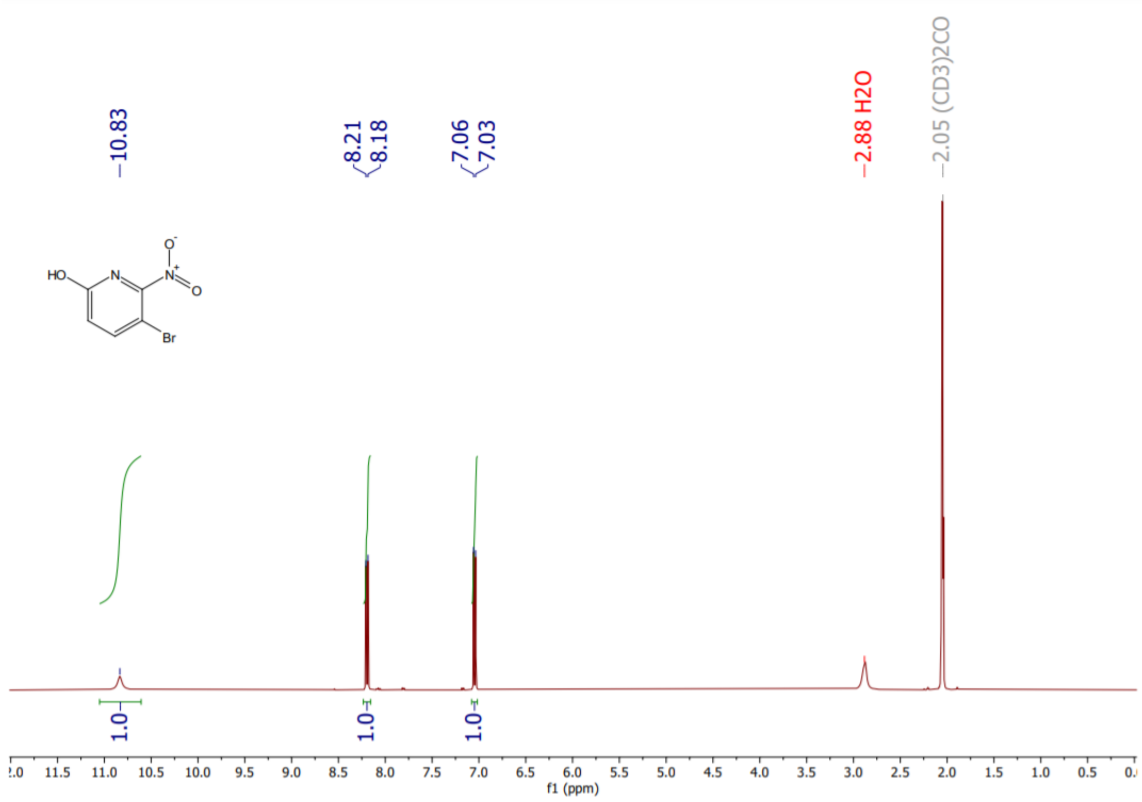

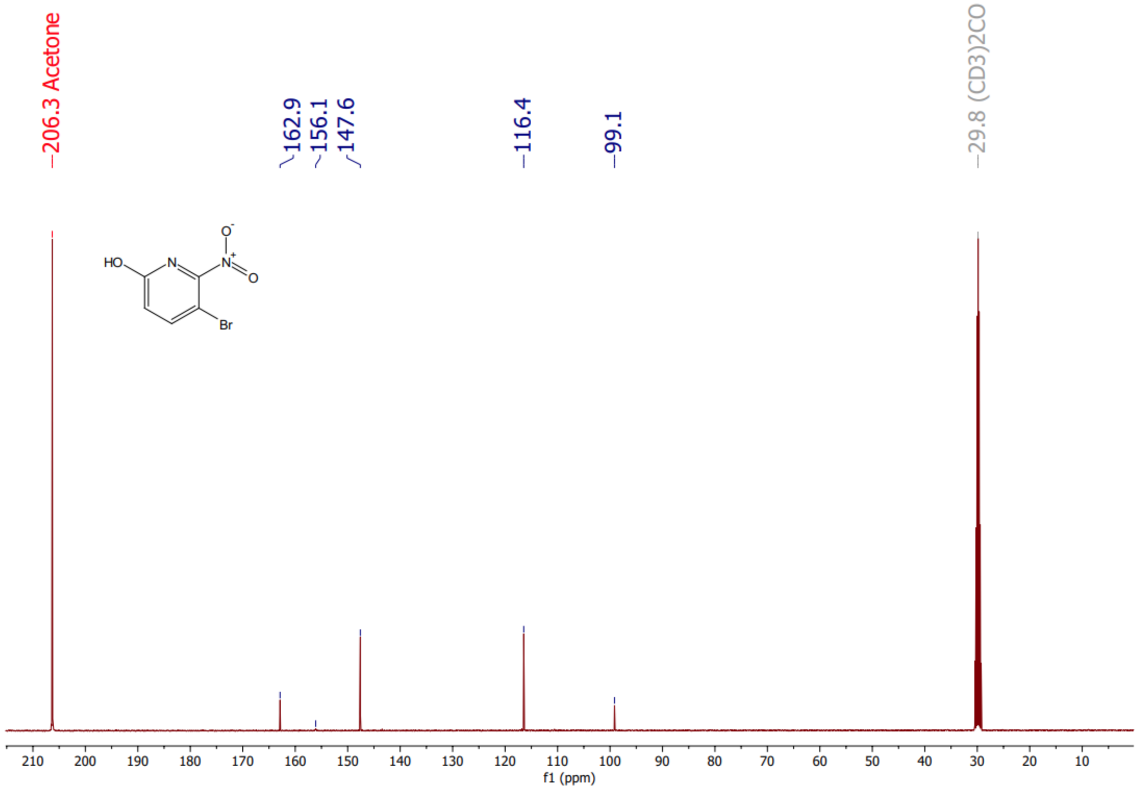

3-Bromo-2-nitro-6-(3-(piperidin-1-yl)propoxy)pyridine

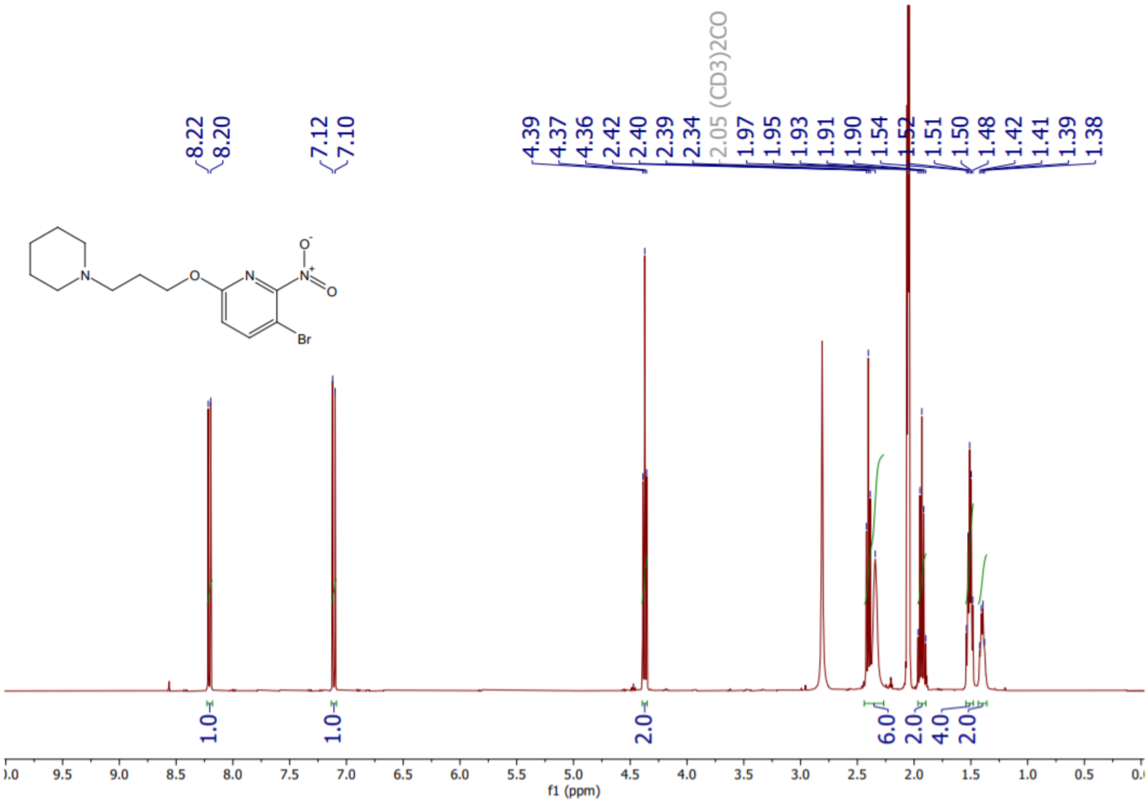

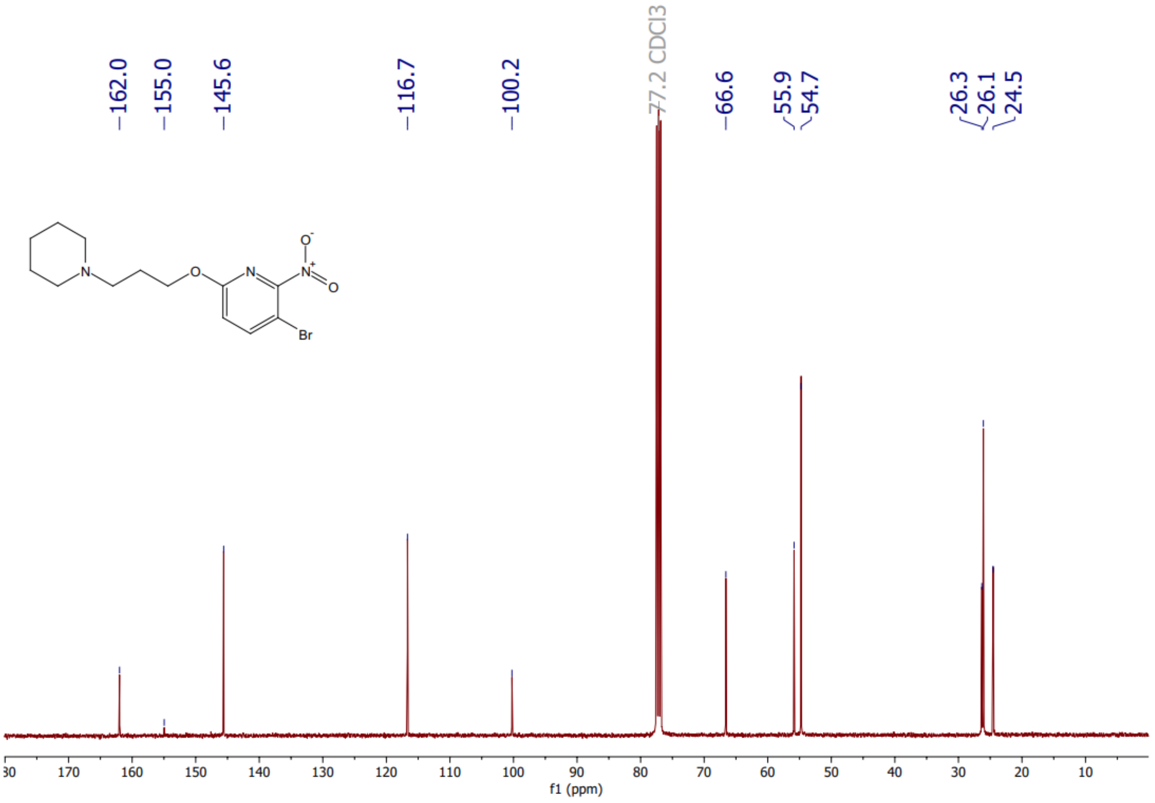

1-Isopropyl-3-methyl-8-(2-nitro-6-(3-(piperidin-1-yl)propoxy)pyridin-3-yl)-1,3-dihydro-2H-imidazo[4,5-c]quinolin-2-one (Precursor 2)

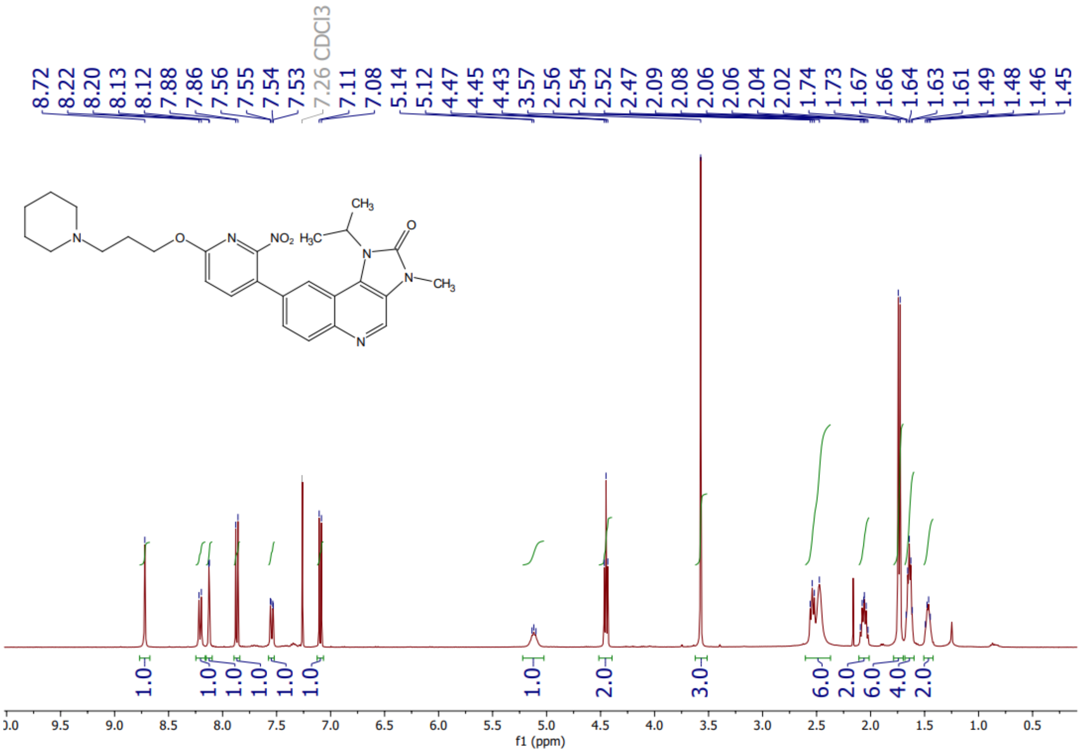

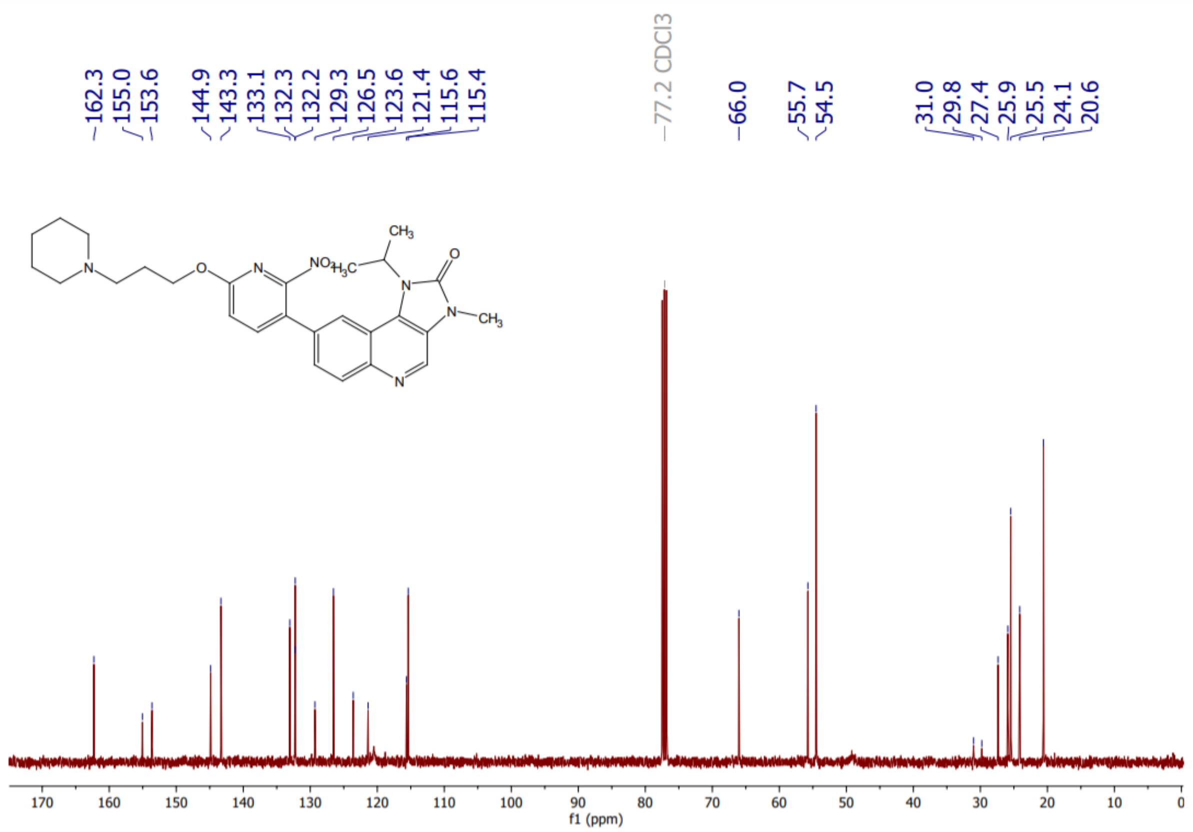

162.3
155.0
153.6
144.9
143.3
133.1
132.3
132.2
129.3
126.5
123.6
121.4
115.6
115.4
77.2 CDCl3
66.0
55.7
54.5
31.0
29.8
27.4
25.9
25.5
24.1
20.6
CH3
O
N
NO2
H3C
N
N
CH3
N
N
O
170
160
150
140
130
120
110
100
90
80
70
60
50
40
30
20
10
0
f1 (ppm)

8. Supporting information references

1. Barlaam, B., Eatherton, A., Hunt, T. and Pike, K., 2017. 8-[6-[3-(amino)propoxy]-3-pyridyl]-1-isopropyl-imidazo[4,5-c]quinolin-2-one derivatives as selective modulators of ataxia telangiectasia mutated (ATM) kinase for the treatment of cancer. WO2017046216A1.

2. Bjork, S., Delisser, V., Johnstrom, P., Nilsson, N. A., Ruda, K., Schou, P. M. and Swahn, B-M., 2010. New Benzofurans Suitable As Precursors To Compounds That Are Useful For Imaging Amyloid Deposits. WO2010024769A1.
